# Genetic Control of Contagious Asexuality in the Pea Aphid

**DOI:** 10.1371/journal.pgen.1004838

**Published:** 2014-12-04

**Authors:** Julie Jaquiéry, Solenn Stoeckel, Chloé Larose, Pierre Nouhaud, Claude Rispe, Lucie Mieuzet, Joël Bonhomme, Frédérique Mahéo, Fabrice Legeai, Jean-Pierre Gauthier, Nathalie Prunier-Leterme, Denis Tagu, Jean-Christophe Simon

**Affiliations:** 1 INRA, UMR1349, Institute of Genetics, Environment and Plant Protection, Domaine de la Motte, Le Rheu, France; 2 INRIA Centre Rennes - Bretagne Atlantique, GenOuest, Campus de Beaulieu, Rennes, France; University of Texas, United States of America

## Abstract

Although evolutionary transitions from sexual to asexual reproduction are frequent in eukaryotes, the genetic bases of such shifts toward asexuality remain largely unknown. We addressed this issue in an aphid species where both sexual and obligate asexual lineages coexist in natural populations. These sexual and asexual lineages may occasionally interbreed because some asexual lineages maintain a residual production of males potentially able to mate with the females produced by sexual lineages. Hence, this species is an ideal model to study the genetic basis of the loss of sexual reproduction with quantitative genetic and population genomic approaches. Our analysis of the co-segregation of ∼300 molecular markers and reproductive phenotype in experimental crosses pinpointed an X-linked region controlling obligate asexuality, this state of character being recessive. A population genetic analysis (>400-marker genome scan) on wild sexual and asexual genotypes from geographically distant populations under divergent selection for reproductive strategies detected a strong signature of divergent selection in the genomic region identified by the experimental crosses. These population genetic data confirm the implication of the candidate region in the control of reproductive mode in wild populations originating from 700 km apart. Patterns of genetic differentiation along chromosomes suggest bidirectional gene flow between populations with distinct reproductive modes, supporting contagious asexuality as a prevailing route to permanent parthenogenesis in pea aphids. This genetic system provides new insights into the mechanisms of coexistence of sexual and asexual aphid lineages.

## Author Summary

Asexual lineages occur in most groups of organisms and arise from loss of sex in sexual species. Yet, the genomic bases of these transitions remain largely unknown. Here, we combined quantitative genetic and population genomic approaches to unravel the genetic control of shifts towards permanent asexuality in the pea aphid, which conveniently shows coexisting sexual and asexual lineages. We identified one main genomic region responsible for this transition located on the X chromosome. Also, our population genetic data indicated substantial gene exchange between these reproductively distinct lineages, potentially leading to the conversion of some sexual lineages into asexual ones in a contagious manner. This genetic system provides new insights into the mechanisms of coexistence of sexual and asexual lineages.

## Introduction

While sexuality is the dominant reproductive mode in metazoans, parthenogenesis - the development of an embryo from an unfertilized egg - occurs in most branches of the animal kingdom (e.g. molluscs, insects, crustaceans, nematodes, fish, reptiles) [e.g. 1,2,3]. Cyclical parthenogenesis (CP) represents a mixed reproductive mode with an alternation of sexual reproduction and parthenogenesis, and is reported in many animal species [4]. The loss of the sexual phase in CP species - leading to permanently parthenogenetic taxa - have been shown to arise from diverse mechanisms, including microbial infection, hybridization, contagion via pre-existing parthenogenetic lineages or spontaneous mutations [5]–[9]. Nevertheless, in case of contagious or mutational origin, the precise genomic regions responsible for the transitions to obligate parthenogenesis (OP) remain largely unknown, mostly because dissecting the genetic bases of that trait using recombination-based approaches is not possible in strictly asexual species. However several species show coexisting CP and OP lineages, with OP lineages often retaining a residual production of males. Such species offer ideal systems to decipher the heredity and therefore the genetic basis of the loss of sexual reproduction. In the rare cases where it has been explored, genetic control of this trait has been shown to be rather simple, with the involvement of one to four loci, depending on the studied organisms [10]–[15]. However, the precise location and underlying function of these genetic factors have not been elucidated.

The ancestral life-cycle of aphids is cyclical parthenogenesis [16], which consists in an alternation of sexual and asexual generations. In spring and summer, CP lineages produce asexual females through apomictic parthenogenesis. In autumn, asexual females give birth to males and sexual females in response to photoperiodic cues (note that CP lineages can also produce asexual females to some extent [e.g. 17]). Sexual females are strict clones of their asexual mothers, while one of the two X chromosomes is randomly lost to generate males [17]. Eggs produced by sexual females are the only frost-resistant stage in the aphid cycle. Hence, a CP life cycle is required to survive in regions with cold winters. In addition, many aphid species also encompass OP lineages which are characterized by an altered response to sex-inducing environmental cues as they produce only asexual females (although they often produce some males [18], [19]). These lineages are thus cold-sensitive because of their inability to lay eggs. Yet, OP lineages are favoured in regions with mild winters where they have a major demographic advantage over CP lineages [20], [21]. Accordingly, CP lineages dominate in cold areas and OP lineages in warmer regions, and both coexist in regions with fluctuating winter temperatures [18]–[20]. Because male production by OP lineages is difficult to prove in the wild, it has been demonstrated in a single study which also showed that these males actually contribute to sexual reproduction with CP lineages [22], [23]. While the switch from clonal to sexual reproduction in CP aphids is triggered by photoperiodic changes, the loss of sexual form production in OP aphids is genetically determined, changes in environmental conditions having little or no effect on their reproductive phenotype [10], [19].

Here, we combined QTL and genome scan approaches to decipher the genetic bases of reproductive mode variation in the pea aphid *Acyrthosiphon pisum*. This species conveniently shows CP lineages (here defined as those able to produce sexual females) and OP lineages (defined as those unable to produce sexual females), and these two types of lineages locally co-occur in regions with intermediate climate conditions [24]. These independent QTL and genome scan approaches outlined the same genomic region as controlling obligate parthenogenesis, this trait being recessive and determined by an X-linked locus. Our data also indicate that asexuality is transmitted in a contagious manner, leading to the conversion of sexual lineages into asexual ones.

## Results

### Genetic maps and QTLs analyses

**Figure 1:**
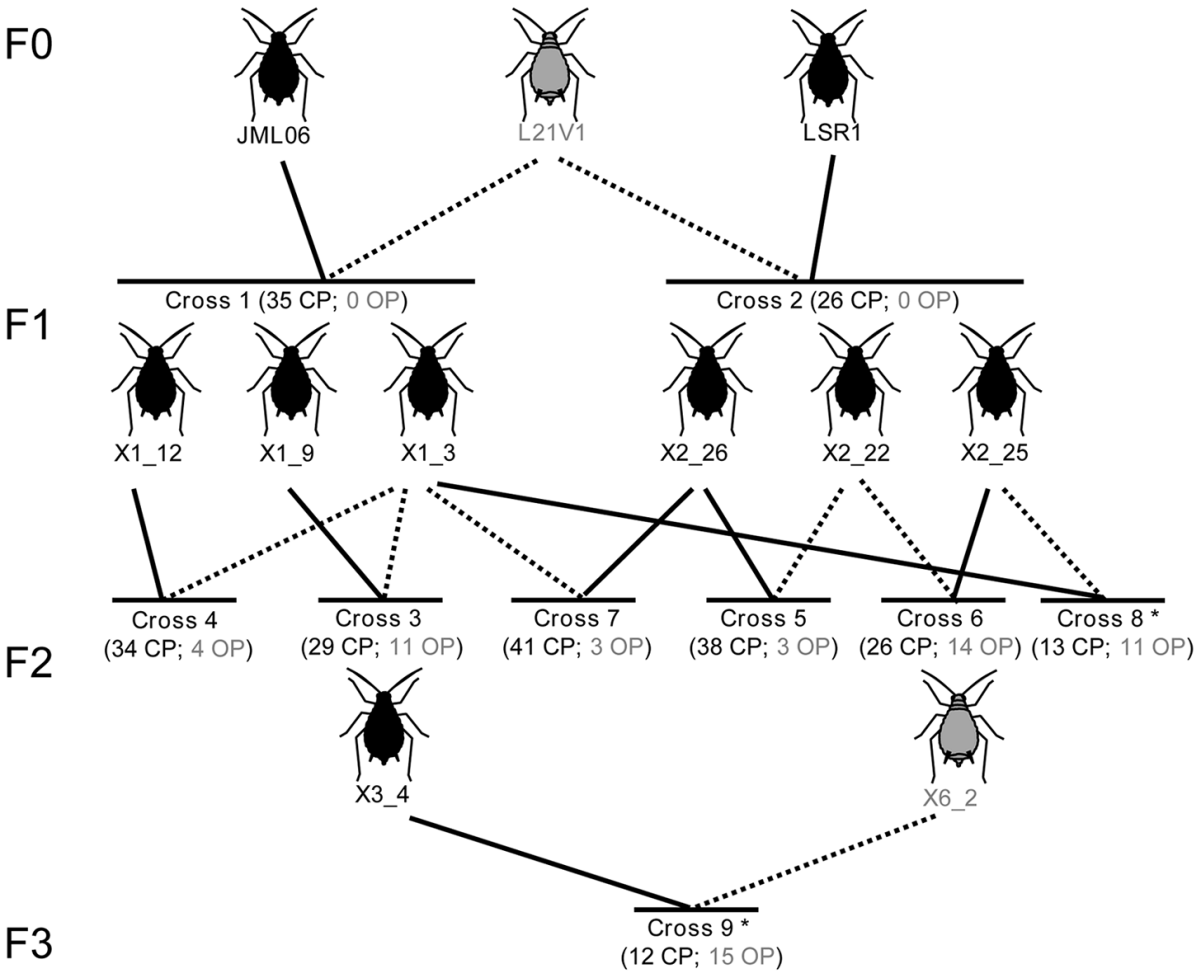
Crossing design and reproductive phenotype of the F1, F2 and F3 progeny. The name of each lineage is shown below the aphid picture and the color of the aphid picture stands for the reproductive phenotype of each lineage used as parent in crossings (grey for obligately parthenogenetic [OP] and black for cyclically parthenogenetic [CP]). The lines show which individuals were crossed (the cross ID is shown below), plain lines indicating that the lineage was used as female and dotted lines, as male. For each cross, the number of progeny determined as CP and OP is also shown. Only crosses 3 to 7 were included in the QTL analyses (crosses 8 and 9 - identified with an asterisk - were not used because progeny was selected according to genotype at the candidate region and was genotyped only at a subset of markers).

**Figure 2:**
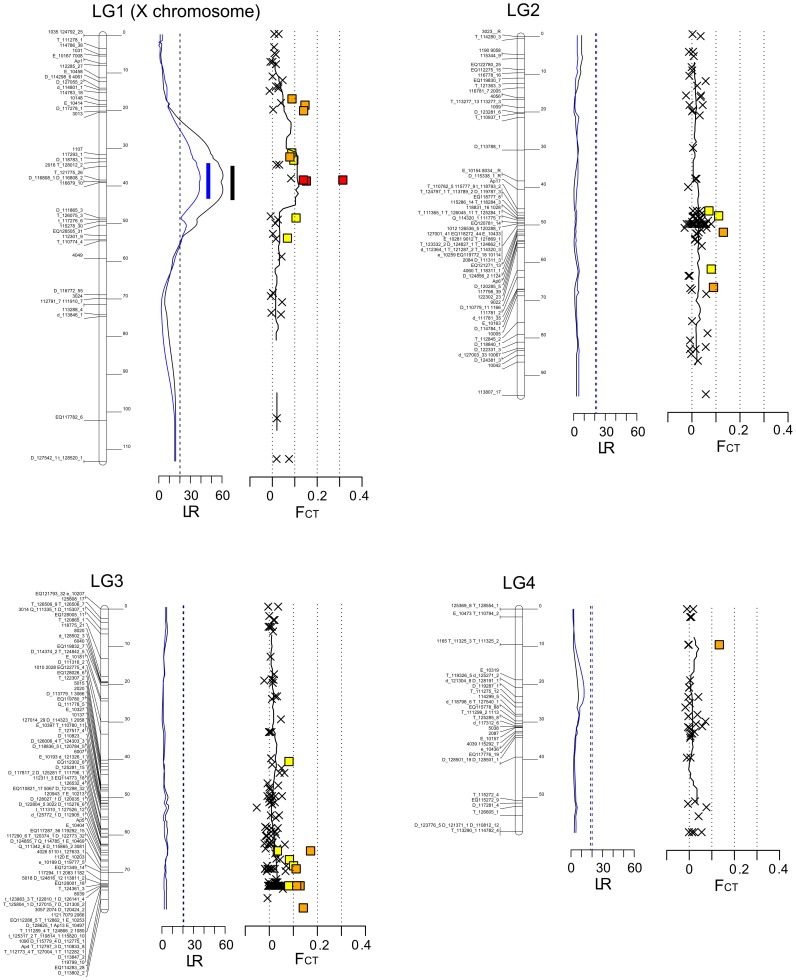
Localization of the genomic region controlling reproductive phenotype on genetic maps of the pea aphid. On these maps of each chromosome (LG1 to LG4), we show results from two independent approaches to identify genomic regions that control reproductive mode variation (i.e. the production of sexual females) in the pea aphid. 1) QTL approach: Likelihood ratio (LR) along chromosomes for the presence of a QTL are shown (solid blue curves correspond to LR values for % of sexual females, and solid black curves to LR values for occurrence of sexual females). The LR thresholds corresponding to a *p*-value of 0.05 at the genome-level (i.e. adjusted for multiple testing) are also shown (dashed blue line for % of sexual females and dashed black line for occurrence of sexual females). Threshold lines for the two traits are almost superimposed. The blue and black bars show the location of the 95% CI of the QTL for % of sexual females and occurrence of sexual females, respectively. 2) Population genomic approach: a hierarchical genome scan was performed using ARLEQUIN 3.5 to identify genomic regions involved in reproductive mode variation. The genetic differentiation values (*F*_CT_) among three OP and three CP populations are shown along chromosomes. Loci identified as significant outliers (hence candidate loci for reproductive mode variation) at 1%, 5% or 10% are shown in red, orange and yellow, respectively, and non-outlier markers with a cross. The black line corresponds to a moving average on *F*_CT_ (calculated on a 15 cM window).

We produced F1 crosses between males of an obligate parthenogenetic lineage (L21V1) and sexual females of two cyclically parthenogenetic lineages (JML06 and LSR1) (Fig. 1). Five F2 crosses (families 3 to 7) involving 6 F1 lineages were performed to obtain a genetic map and to locate QTL controlling the presence and proportion of sexual females by genotypes placed under sex-inducing conditions. A total of 305 microsatellite markers (out of 394) was successfully ordered on the genetic maps. These loci clustered in four linkage groups that correspond to the four chromosomes of the pea aphid [25]. 45 loci locate on the X chromosome (LG1 following notation in [26]), and 85, 135, and 40 on LG2, LG3 and LG4, respectively. Average map length (over males and females) was 113, 95, 79 and 59 cM for LG1, LG2, LG3 and LG4, respectively (Fig. 2). Of the 89 unmapped loci (out of 394), 51 were monomorphic in the 3-generation pedigree, five were homozygous in all F1 females, and 33 showed null alleles at high frequencies or inconsistent genotypes (presumably due to difficulties to score alleles).

By contrast with the 61 F1 progeny which all produced sexual females (hence were classified as CP) segregation of reproductive phenotype was observed among the five F2 families (Fig. 1, families 3 to 7). All five families (203 F2 genotypes) comprised a mixture of genotypes expressing either an OP (no sexual females produced at all) or a CP (sexual female production ranged from 22% to 77%) phenotype. The percentage of OP F2 ranged from 7% to 35% depending on families (Fig. 1, see also S1 Figure). Contrastingly, 97% of F2 lineages produced males: only 5 out of 35 OP lineages, and 2 out of 168 CP lineages did not produce males (S1 Figure). QTLs analyses on these five F2 families revealed one candidate genomic region located on the X chromosome (LG1) for the control of reproductive mode variation (measured as the proportion of sexual females or occurrence of sexual females), as evidenced by likelihood-ratio (LR) values for these traits above the LR thresholds corresponding to the null hypothesis of no QTL. The QTL for the proportion of sexual females produced locates at 38.0 cM on LG1 based on highest LR values. The 95% confidence interval [CI] for QTL position is 34.0–43.2 cM (Fig. 2). The QTL for presence/absence of sexual females locates at 37.6 cM on LG1 and the 95% CI is 34.8–43.6 cM (Fig. 2). We then accounted for the presence of a QTL at position ∼38 cM to test for a second QTL (see Methods). No significant support for a second QTL was found for either traits, as LR values along the four chromosomes were largely below the LR thresholds corresponding to 5% significance at the genome level.

We then focused on the genomic region pinpointed by the QTL analysis (∼38 cM on LG1) and looked at the alleles inherited by F2 individuals. In three F2 families (families 3, 4 and 6, Table 1), the 29 F2 lineages that expressed an OP phenotype had the same genotype as the OP lineage L21V1 (F0) at all markers located on the X-chromosome between T_128012_2_G (34.8 cM) and T_126075_3_Y (49 cM). For simplification, we refer to this multilocus genotype as “*op1/op2*” (Table 1, see also S1 Figure). Contrastingly, the 89 CP F2 individuals from families 3, 4 and 6 all possessed at least one allele inherited from their CP grandmothers in this genomic region (the four possible alleles from the two CP grandmothers are collectively referred to as “*CP*”). Hence these individuals were either *op1/CP*, *op2/CP* or *CP/CP* (Table 1, S1 Figure). In these three F2 families, the *op1* allele was transmitted through the F1 fathers from the OP grandfather (L21V1, of genotype *op1/op2*). Since chromosomes in male pea aphids do not recombine [27], the entire X-chromosome of grandfather L21V1 that carries the *op1* allele was transmitted to its grandchildren. Conversely, the *op2* allele was inherited from the F1 mothers, which themselves inherited the whole *op2*-bearing X-chromosome from their OP grandfather. Recombination of the *op2*-bearing X-chromosome in the F1 mothers allowed reducing the region controlling reproductive modes between markers T_128012_2_G (34.8 cM) and T_126075_3_Y (49 cM) on the *op2*-bearing X chromosome (S1 Figure). Based on the results from QTL analyses, we performed two additional crosses. Here only a subset of individuals per cross were phenotyped (24 and 27, respectively), chosen according to their genotype at 8 microsatellite markers in the genomic region of interest. A F3 cross (cross 9, see Fig. 1 and Table 1) confirmed the location of the QTL and allowed further narrowing down its upper boundaries to marker 111865_3 [48.5 cM] (see S1 Figure). We finally crossed *op1/CP2* females with *op2/CP3* males in order to recombine the *op1*-bearing X-chromosome (cross 8, Table 1). All the 11 lineages that harboured the *op1* allele in combination with the *op2*-bearing X-chromosome were OP, and recombination in the *op1*-bearing X-chromosome showed that the region controlling reproductive phenotype lies between markers 116879_10 (39.1 cM) and D_111865_3 (48.5 cM) on the *op1*-bearing X copy (see S1 Figure). These different crosses revealed that *op1* and *op2* alleles are recessive over *CP* alleles (since the 76 *op1/CP* and the 41 *op2/CP* lineages are CP, and the 12 *op2/op2* and 43 *op1/op2* lineages are OP, Table 1). Noteworthy we observed that *op1/op1* genotypes can have either a CP (11 lineages) or an OP (6 lineages) phenotype (Table 1), suggesting that other genetic or environmental factors mitigate the control of reproductive phenotype in lineages *op1/op1* at the major candidate locus.

**Table 1:** Reproductive phenotype of F2 and F3 individuals according to their genotype in the candidate region.

Genotype	Families							Overall
	cross 3 ♂*op1/CP2*×♀*op2/CP1*	cross 4 ♂*op1/CP2*×♀*op2/CP1*	cross 5 ♂*op1/CP4*×♀*op1/CP3*	cross 6 ♂*op1/CP4*×♀*op2/CP3*	cross 7 ♂*op1/CP2*×♀*op1/CP3*	cross 8^a^ ♂*op2/CP3*×♀*op1/CP2*	cross 9^a^ ♂*op1/op2*×♀*op2/CP2*	
*op2/op2*	-	-	-	-	-	-	12/12 **(100%)**	12/12 **(100%)**
*op1/op2*	11/11 **(100%)**	4/4 **(100%)**	-	14/14 **(100%)**	-	11/11 **(100%)**	3/3 **(100%)**	43/43 **(100%)**
*op1/op1*	-	-	3/10 **(30%)**	-	3/7 **(43%)**	-	-	6/17 **(36%)**
*op1/CP*	0/5 **(0%)**	0/10 **(0%)**	0/18 **(0%)**	0/14 **(0%)**	0/19 **(0%)**	0/5 **(0%)**	0/5 **(0%)**	0/76 **(0%)**
*op2/CP*	0/10 **(0%)**	0/14 **(0%)**	-	0/7 **(0%)**	-	0/3 **(0%)**	0/7 **(0%)**	0/41 **(0%)**
*CP/CP*	0/14 **(0%)**	0/10 **(0%)**	0/13 **(0%)**	0/5 **(0%)**	0/18 **(0%)**	0/5 **(0%)**	-	0/65 **(0%)**

Given are the number of lineages determined as OP, the number of lineages phenotyped, and the percentage of OP lineages (in brackets). *op1* and *op2* alleles correspond to the alleles inherited from the L21V1 grandparent clone (OP phenotype). The four different alleles inherited from the two CP grandparents JML06 (alleles *CP1* and *CP2*) and LSR1 (*CP3* and *CP4*) were aggregated as “*CP*” since we did not observe differences of reproductive phenotype for the four different CP alleles (see Fig. S1 for detailed information for each allele).

a Progeny in these crosses was selected based on genotype at the candidate locus.

### Genome scans of wild OP and CP populations of the pea aphid

A 436-marker genome scan performed on 109 individuals from wild populations collected in environments selecting for different reproductive modes (OP or CP) revealed four loci having excessive genetic differentiation (*F*_CT_) at the α = 0.01 threshold (ARLEQUIN 3.5 analysis, Table 2, S2 Figure). *F*_CT_ between populations under selection for different reproductive modes ranged from 0.14 to 0.31 at these four outlier loci, while the median *F*_CT_ value estimated over the 436 markers was 0.014 (average 0.025). Among these four outliers, T_111491_2 was also identified as outlier under balancing selection in the populations from CP-selecting environment (*F*_ST_ among CP populations was 0.0003, and *He* 0.56) when ARLEQUIN analyses were performed among populations assumed to share the same reproductive mode (Table 2). This locus was not successfully genotyped in the families so its genomic location remains unknown. Interestingly, the three other outliers co-locate on the X-chromosome and within the same genomic region identified with the experimental (QTL) approach (Fig. 2). Accordingly, *F*_CT_ values along the genetic map of the four chromosomes show a clear peak of genetic differentiation in the QTL region (Fig. 2). In this region, expected heterozygosity in OP populations was lower than in CP populations (S3 Figure), while heterozygosity values of the three CP populations and the three OP populations were similar along other regions of the chromosomes.

**Table 2:** Outlier loci identified by genome scans.

Locus ID	*F_CT_* between OP and CP populations	*p*	*H_E, overall_*	*F_ST_* between CP populations	*p*	*H_E,CP_*	*F_ST_* between OP populations	*p*	*H_E,OP_*	Chromosome	Position on chromosomes (cM)
D_116808_1	0.31	0.00001	0.86	0.085	0.09	0.60	0.01	0.17	0.58	X	38.9
D_116808_2	0.14	0.008	0.83	0.012	0.15	0.86	−0.01	0.21	0.57	X	38.9
116879_10	0.15	0.005	0.84	0.066	0.10	0.81	0.06	0.16	0.61	X	39.1
T_111491_2	0.22	0.008	0.41	0.00003	0.0004	0.56	0.04	0.21	0.07	Unknown	Unknown

Outlier loci detected with ARLEQUIN 3.5 at α = 0.01 in a hierarchical analysis in which geographical populations were nested within group of populations experiencing selection for the same reproductive mode (OP *vs* CP). *F_CT_* between CP and OP populations (and significance as outlier) are shown, as well as expected heterozygosity (*H_E_*). Outlier detection analyses were also performed among OP and CP populations to ensure these loci were not outlier at this hierarchical level. The position on chromosomes is also given.

## Discussion

We have shown here that a key ecological trait – the variation in reproductive mode – was controlled by one main genomic region in the pea aphid. This ∼9 cM-wide X-linked region was identified by two independent and complementary approaches: the co-segregation of molecular markers and phenotypes in experimental crosses and a large scale population genomic survey (genome scans). Interestingly, 100% of phenotypic variation was explained by the genotype at the candidate locus in five crosses (crosses 3, 4, 6, 8, 9, Table 1). In the two remaining families (crosses 5 and 7), this genomic region was also strongly associated with reproductive phenotype (as all six OP F2 were *op1/op1* at this candidate region) but linkage was not absolute (as 11 *op1/op1* individuals are nevertheless CP) (Table 1). Two hypotheses can be invoked for this lack of association in some F2 genotypes. First, an additional locus with minor effects might contribute to the control of reproductive mode variation, its contribution being only visible in individuals *op1/op1* at the major locus (all 68 individuals from crosses 5 and 7 that are not *op1/op1* are CP). A second hypothesis is that the production of sexual females depends on a threshold concentration of some unknown factor (e.g. transcript, protein, hormone). Under this assumption, minor environmental variation could have drastic effect on reproductive phenotype determination around the concentration threshold. We tested for the presence of a second QTL (first hypothesis), and found no statistical support for it. Yet, power to detect such an additional QTL was low (due to the small sample size of *op1/op1* genotypes) so we cannot at the moment disentangle these two hypotheses. Nevertheless, the mostly single-locus recessive inheritance of obligate parthenogenesis in the pea aphid is in line with the few similar studies which showed that the transition from sexual to asexual reproduction is determined by a small number of loci [10]–[14].

Transitions from cyclical parthenogenesis (CP, i.e. the alternation of asexual and sexual generations) to obligate parthenogenesis (OP) in aphids probably occur through loss-of-function mutations leading to an inability of lineages to produce females in response to the environmental cues that normally trigger the sexual phase. Hence, any mutation (i.e. point mutation, indel or rearrangements) that disrupts the pathway leading to the production of sexual females might be responsible for this transition. In theory, these loss-of-function mutations could occur repeatedly in the same gene, or on different genes involved in the same molecular cascade, these genes being either neighbours or scattered on the genome. Herein, the OP grand-parent used for QTL mapping harbours two distinct alleles (*op1* and *op2*) at the identified QTL and the phenotype of homozygotes *op2/op2* and *op1/op1* significantly differs (all 12 *op2/op2* but only 6 of the 17 *op1/op1* individuals are OP, test of proportion: *p* = 0.0016). This indicates that at least two independent mutations in the same region are involved in the loss of sexual reproduction. Remarkably, the genome scan demonstrates that the region identified by the QTL approach also shows signatures of divergent selection between populations under different selective regimes for reproductive mode. This indicates that the QTL identified with three laboratory clones is also involved in the control of reproductive mode in wild populations originating from a large-scale geographic area (populations were collected up to 700 km apart). These population genomic data give further insights into the transitions from CP to OP. In particular, the occurrence of outliers in the QTL region, combined with their low genetic diversity in OP- compared to CP-selecting environments, reveal that only one or a few mutations leading to the OP phenotype have reached high frequencies in OP-selecting environments (otherwise this genomic region would not have been identified as *F*_ST_-outlier). Outside the candidate region, populations from CP- and OP-selecting environments are weakly differentiated and show highly correlated levels of genetic diversity along chromosomes, suggesting important gene flow.

The most likely scenario to explain these genomic patterns of differentiation involves bidirectional gene exchanges between CP and OP lineages: Let us consider that the rare males produced by OP lineages successfully mate with sexual females from CP (as it is the case in laboratory conditions and presumably into the wild), producing CP offspring heterozygous at the candidate region (*op/CP*). These heterozygous CP lineages may produce OP progenies (those homozygote for the *op* alleles) that would survive if they encounter mild winter environments. Some minimal amount of gene flow can maintain a low genetic differentiation between populations from OP- and CP-selecting environments at the scale of the genome since divergence for neutral loci at a migration drift equilibrium is prevented when *Nm*>1, *N* being the effective population size and *m* the migration rate [28], [29]. Such bi-directional gene flow between OP and CP lineages may occur in the geographical regions with intermediate winter conditions where both CP and OP lineages coexist [22], [30]. Another scenario to consider relies on unidirectional gene flow from CP to OP. Under the hypothesis that recessive *op* alleles are relatively frequent in CP populations, CP lineages will regularly produce new OP lineages (those homozygous at the *op* alleles). If such OP linages are generated frequently, low differentiation between populations from OP- and CP-selecting environments along the genome is expected, except at the candidate region. This scenario is however less parsimonious than the former. First, it requires very frequent production of OP lineages by CP ones in order to prevent genomic differentiation between these two compartments likely to result from the strong clonal fluctuations (due to neutral factors and/or selection) typical of asexual populations [31], [32]. Second, in absence of reciprocal gene flow from OP to CP lineages, positive selection on *op* alleles in CP populations should be invoked to maintain these alleles. Yet, we know that *op* alleles are associated with a cost in CP selecting environments (homozygous *op/op* individuals do not survive cold winters) and therefore their frequencies are expected to decrease under these conditions. Our data are thus best explained by bidirectional gene flow between populations of distinct reproductive modes and support the hypothesis of contagious asexuality in wild pea aphid populations.

Contagious asexuality has important consequences on the evolvability of the OP lineages. Indeed, the bi-directional gene flow between CP and OP lineages allows genomes and alleles evolved under an asexual regime to enter the “sexual” pool via the few males produced by OP clones. Once introgressed in a CP lineage, a genomic region evolved under an asexual regime will recombine, allowing the purging of deleterious alleles. Later, if some of the CP individuals produce OP progeny (those homozygous at the *op/op* genomic region), some of the alleles evolved under the asexual regime might then reintegrate an OP lineage. Hence, contagious asexuality has the potential to combine the beneficial effects of sex (purging of deleterious mutations and combination of beneficial mutations within the same genome [33], [34]) with the advantages of clonal reproduction that avoid the two-fold cost of sex [34] and can “freeze” a genome (avoiding recombination load) [35]. This genetic system could thus favour the regular emergence of well fit OP lineages, which would be so fit because they would reuse alleles that competed and evolved under an OP-selecting environment (during their long stay within OP lineages) and that would have been separated from linked deleterious mutations during their sojourn in CP lineages.

The physical size of the ∼9 cM candidate region, that represents ∼2.6% of the whole genome in term of recombination units (cM), is still unknown because scaffolds from the pea aphid genome sequence are not yet ordered on chromosomes [36]. Hence the exact number and nature of the genes that are comprised within the candidate region are not known. Nevertheless, already 66 genes encoding proteins have been identified in the three scaffolds covering part of the 9 cM genomic region of interest (S1 Table). It is too early to designate candidate genes responsible for the CP/OP phenotypes, mostly because half of them have no predicted functions. However, recent works on the genetic programs involved in the seasonal switch from clonal to sexual reproduction in CP lineages allow highlighting in the candidate region three predicted genes putatively involved in photoperiod perception and brain signalling (e.g. rhodopsin specific isomerase, insulin), two pathways identified as differentially expressed in aphids exposed to either clonal or sex induction regimes [37]. Two genes putatively involved in the melavonate pathway (farnesyl-pyrophosphate synthase like and hydroxymetharyglutaryl-CoA synthase) upstream of the juvenile hormone synthesis, which is known as being a key regulator of reproductive orientation in aphids [38], [39], also locate within the candidate region.

To conclude, here we combined population genomics and quantitative genetics to identify the genetic bases of a key trait for aphid adaptation to climate - the loss or maintenance of sexual reproduction. We found this trait to be controlled by one main genomic region located on the X chromosome. The widespread geographical distribution of a few alleles associated with obligate asexuality suggests that these alleles might be particularly advantageous for OP lineages, and might have outcompeted previously established *op* alleles, a hypothesis that deserves further investigations.

## Materials and Methods

### Experimental crosses and assessment of reproductive phenotype

We crossed individuals from three genotypes (clones LSR1, L21V1, JML06) that present contrasted reproductive phenotypes. These three F0 lineages were chosen based on their ability to produce or not sexual females under standard sex-inducing conditions (i.e. short photoperiod with 12 h light) [37]. All aphids were reared on *Vicia faba* (broad bean) because it is a universal host for all known host races of the pea aphid species complex [40], [41] and also because this plant is easier to grow compared to *Medicago* ssp. LSR1 (collected on *Medicago sativa* in New-York, USA in 1998 and used for complete genome sequencing [36]) produces males (21%), sexual females (54%) plus some parthenogenetic females (25%) under standard sex-inducing conditions. Under the same inducing conditions, JML06 (sampled on *Medicago lupulina* in Jena, Germany in 2006) produces only sexual individuals (70% males and 30% sexual females). Contrastingly, L21V1 (sampled on *M. sativa* in Rennes, France in 2003) produces only parthenogenetic females (89%) and a few males (11%). LSR1 and JML06 are therefore classified as cyclical parthenogens (CP) and L21V1 as obligate parthenogen (OP). Crosses between males from the OP and (sexual) females from the CP lineages were performed. For this, one L4 larva from each of the three grandparent clones was moved to a new broad bean plant and transferred to a climatic chamber with a 12 h photoperiod (18°C) to trigger the production of sexual females and males in CP lineages (and males in the OP lineage) [37]. Then, for each lineage, three larvae of the next clonal generation were isolated on three different broad bean plants. Once the larvae reached adulthood and started to give birth to nymphs, groups of 10 larvae of the next generation were isolated on new broad bean plants until the asexual female stopped reproducing and died. The morph of each individual of this second clonal generation (i.e. male, sexual females, asexual females) was determined at adult stage based on morphological characters (males are slender than females, and the legs of sexual females are longer that those of asexuals). The few individuals that died before reaching the adult stage (hence before being sexed) were also counted. Then a total of 50 males from the clone L21V1 and 50 sexual females from the clone JML06 were put together on broad bean plants (*Vicia faba*) to generate a F1 family (cross 1: JML06 ♀×L21V1 ♂, Fig. 1). The 50 sexual females used in the cross are clonal. However, males consist of two different genotypes because they inherit randomly one of the two X copies from their asexual mother (approximately half of males are expected to bear the first X copy of their mother and the second half the other copy) [17]. A second F1 family was generated similarly by crossing 50 L21V1 males with 50 LSR1 sexual females (cross 2, Fig. 1). In Fig. 1, dotted lines show lineages used as male and plain lines those used as female. Eggs were kept at 4°C (80% humidity) for 85 days and were then transferred at 18°C for hatching. A few days after the first eggs hatched, 50 parthenogenetic larvae for each cross were isolated on new broad bean plants. Each F1 lineage was kept for 7 to 9 months under conditions sustaining clonal reproduction (16 h light, 18°C). Reproductive phenotype of the F1 lineages was then assessed similarly.

Six F1 lineages (three per cross) were then chosen to produce the next F2 generation (Fig. 1). All F1 produced sexual females (with from 27% to 71% and 28% to 64% sexual females for cross 1 and 2, respectively), hence were CP. The 6 F1 clones were thus chosen to cover the diversity in terms of the production of males (that ranged from 0–73% and 0–55% males for cross 1 and 2, respectively) and asexual females (that ranged from 0–42% and 1–53% for cross 1 and 2, respectively). Five F2 crosses (crosses 3 to 7 in Fig. 1) were performed using the same protocol as for the F1. 44 to 47 F2 lineages per family (hence 229 F2 lineages in total) were then isolated and kept for subsequent assessment of reproductive mode phenotype (same protocol as for the F0 and F1). Twenty-six F2 lineages (out of 229) died before being phenotyped.

### Genotyping, genetic map and detection of QTLs

The three grand-parents, the six F1 parents and the 229 F2 individuals from families 3 to 7 were typed at 401 microsatellite loci (see S2 Table for loci used and [42]–[44] for primer sequences). We first checked for the presence of null alleles by looking at the inheritance of alleles in the 3-generation pedigree. Homozygous individuals originating from parents displaying a null allele were transformed into missing data. Loci located on the same chromosome were identified based on their complete linkage in males (2n = 8 in the pea aphid and chromosomes in males do not recombine) [27], [45]. Genetic maps were then constructed for each of the four chromosomes with Crimap 2.53 [46] using Kosambi mapping function. Linkage maps were drawn using MAPCHART v. 2.1 [47]. QTL detection was then performed with the interval mapping method implemented in QTLmap, using the LDLA approach [48]. The phenotypic traits analysed for each F2 lineage (from crosses 3 to 7) were the occurrence (binary variable) and the percentage (quantitative variable) of sexual females in the parthenogenetically produced offspring. We focused on these traits because the production of sexual female is the most relevant variable to predict whether a population is able to reproduce sexually or not [10], [49]. In our analyses, we set parameter *ndmin* to 200 so that no information from males meioses was used to locate QTLs (since chromosomes do not recombine in males, males are not informative to locate QTLs within chromosomes). QTLs were detected based on likelihood-ratio (LR) along chromosomes. LR values corresponding to a significance level of 0.05 for each chromosome were empirically determined from 1,000 simulations under the null hypothesis of the test (i.e. no QTL). Genome-wide significance levels (i.e. LR values corresponding to adjusted *p*-values) were computed to account for multiple testing (i.e. four tests, corresponding to the four chromosomes). The drop-off method implemented in QTLmap was applied to obtain 95% confidence intervals of the QTL location. Similarly to the reduction of x-LOD when using LOD scores, the maximum LR value was reduced by 3.84 (corresponding to a Chi^2^ distribution with one degree of freedom for *p*<0.05) to determine a threshold. Region boundaries were then defined by the LR locations crossing this threshold upstream and downstream of the peak LR [as described in 50], [51] to identify the 95% CI of the QTL. After identifying the first QTL (see Results), we tested for the presence of a second QTL. For this, genotype at locus T_121775_26 (the closest marker from the peak of the QTL on LG1) was introduced as a fixed effect in the model. This marker is highly discriminative as each of the three grandparents possesses different alleles. LR for the presence of a QTLs against the hypothesis of no QTL was then compared to LR thresholds corresponding to a 5% significance determined by 1000 simulations in QTLmap.

### Dominance of alleles controlling reproductive mode

The QTL approach led to the identification of a single genomic region, located on the X chromosome, which controls reproductive mode (see Results). Yet, in the three F2 crosses that were highly informative, all lineages used as mother inherited by chance the same X chromosome copy from their OP father (remind that chromosomes do not recombine in male aphids). From these crosses and from recombination events, we determined that the gene(s) that control(s) reproductive mode locate(s) between markers T_128012_2_G (34.8 cM) and T_126075_3_Y (49 cM) on this X copy (see Results). The segment of this X chromosome copy is referred to as “*op2* allele” hereafter. However, we had little power to test whether the same region also controls this trait on the second X copy from the OP grandparent (that we refer to as “*op1* allele”). We therefore performed an additional F2 cross (cross 8: X2_25♂×X1_3 ♀) to recombine the X-chromosome bearing the *op1* allele (the mother X1_3 ♀ bears one *op1* allele). We also crossed X6_2♂×X3_4♀ (that each possesses an *op2* allele, cross 9) to produce homozygous individuals at this X chromosome region (i.e. *op2*/*op2*) to assess the dominance status of the different alleles in the candidate region (i.e. *op1*, *op2*, and those inherited from CP clones, referred to as *CP1*, *CP2*, *CP3* and *CP4*). Since these two crosses were performed after we had identified the genomic region controlling reproductive mode variation, only a subset of individuals were phenotyped (24 and 27, respectively), chosen accordingly to their genotype at 8 microsatellite markers in the genomic region of interest (see S1 Figure for markers used).

### Genome scan approach

Pea aphid individuals were collected in alfalfa fields from six sampling sites (S3 Table). All *A. pisum* individuals were sampled from the same plant species (*Medicago sativa*) to prevent confounding effects of plant or reproductive mode specialization on genetic divergence [52]. Three of the sites locate in north-east France or Switzerland and correspond to regions with cold winters (“temperate continental climate” as defined in [53]). In these areas, pea aphid populations consist mainly of CP lineages, because eggs are the only stage that survives cold winters [19] (we thus consider these areas as CP-selecting environment). Individuals were collected in spring 2008, a few weeks after egg hatching to maximize the probability to sample locally overwintering populations (these samples have been used in [43], [44]). The three other sampling sites locate in south-west France, and correspond to regions characterized by mild winters (i.e. “temperate oceanic climate” as defined in [53]). These areas are considered as OP-selecting environment. Here, sampling took place in winter 2008–2009 because at this season, OP lineages can be discriminated from CP ones, since the former overwinter as parthenogenetic females while the latter spend winter as eggs. Parthenogenetic females were collected from the six populations (see [43] for further details). To obtain sufficient amounts of DNA for genotyping hundreds of microsatellites, field-collected aphids were grown individually in controlled conditions ensuring continuous clonal reproduction (16 h light/day, 18°C). In each of the 6 geographic populations we then kept 20 individuals (except for one population for which only 15 individuals successfully established in the lab to provide sufficient DNA) on which all further analyses were conducted. These 115 individuals were genotyped at 443 microsatellite loci (301 of them were positioned in the genetic maps, see S2 Table). Six individuals (out of 115) with more than 15% missing genotypes were removed as well as seven markers (out of 443) with more than 30% missing data.

To detect loci that depart from neutral expectation, and which are therefore potentially involved in reproductive mode variation, we used a hierarchical method [54] implemented in ARLEQUIN 3.5 [55]. The distribution of the genetic differentiation among populations characterized by different reproductive mode expected under neutrality was estimated by means of coalescent simulations. The among-reproductive mode differentiation was characterized by the parameter *F_CT_*, which accounts for the geographical structure within populations (three populations originate from OP-selecting environments and the three other from CP-selecting environments). 100 000 coalescent simulations were performed conditionally on the multilocus estimate of *F_CT_* at the 436 microsatellite loci, assuming 50 groups and 100 demes per group. The observed data from each locus were compared with the simulated distribution, and a particular locus was classified as a significant outlier if it fell outside the 99% confidence envelope. We focused here on loci putatively involved in divergence between populations with contrasted reproductive mode; hence, we considered in this analysis only the loci falling above the upper confidence limit. As we were interested in identifying outlier loci involved in the variation of reproductive mode, and not in adaptation to local environmental conditions, we checked that the outliers identified from this global analysis (in which the two types of populations were included simultaneously) were not classified as outliers (either under divergent or balanced selection) among populations sharing the same reproductive phenotype. To that end, we ran two independent analyses for the detection of outliers within populations sharing the same reproductive mode. We also checked that the outcomes of genome scan analysis were not affected by the inclusion of markers with >10% missing data (58 loci). Since the confidence interval was similar when including or not markers with >10% missing data and because our aim was to screen the genome with the highest number of markers, we present only the analysis based on the whole dataset (436 markers).

## Supporting Information

Figure S1 Phenotype and genotypic data from the 4-generation pedigree. For each of the 263 F0, F1, F2 or F3 lineage, we show the percentage of sexual females (SF), males (M) and asexual females (AF) produced when the lineage is placed in environmental conditions known to induce the production of sexual forms in cyclically parthenogenetic (CP) lineages. The fecundity (sum of the number of males, sexual females and asexual females produced) and the number of individuals that died before reaching the adult stage (i.e. that were not phenotyped) is also given. These values represent average over three replicates for each lineage (in all cases the three replicates gave the same response regarding the presence or absence of sexual females). Lineages were classified according to the production of sexual females (no sexual female produced: obligate parthenogenetic lineage [OP, showed in pink], sexual female produced: cyclical parthenogenetic lineage [CP]). We also show for each individual its genotype around the genomic region that contains the candidate locus for the production of sexual females (the 95% CI for the QTL ranges from ∼34 cM to ∼43 cM, and the portion of the X chromosome shown here span from 32 cM to 54 cM). Genotypes were phased and each of the different grandparental haplotype (*op1*, *op2*, *CP1*, *CP2*, *CP3* and *CP4*) is shown with a different colour. The upper haplotype (in the F1 to F3 generations) corresponds to fragment of chromosome inherited from the father (i.e. without recombination since male aphids do not recombine). The lower haplotype was inherited from the mother (recombination might occur). Numbers correspond to the size of the allele at each microsatellite marker. Loci not successfully genotyped are indicated as 0 (in grey). Crosses 8 (F2) and 9 (F3) were performed after the identification of the candidate region, with the aims of further validation and to investigate the dominance of the two OP alleles (*op1* and *op2*). Hence, only a subset of loci surrounding the QTL was genotyped in these two crosses (markers 1107, D_118783_1, T_128012_2, T_121775_26, D_116808_1, 116879_10, D_111865_3, 112301_9). These crosses confirmed previous results and allowed to further narrow down the candidate region to the portion of chromosome located between markers 116879_10 (38.9 cM) and D_111865_3 (48.5 cM) based on individuals X8_52 and X8_96). We inferred the putative genotype of each individual at this candidate locus (see the black rectangle and also column “genotype”) by minimizing the number of recombination. In cases where recombination occurred between the markers flanking the candidate region, we considered the phenotypic data to determine the most probable genotype assuming that only *op1/op2* and *op2/op2* genotypes lead to obligate parthenogenesis (e.g. see F2 lineage X6_45, cross 6). When the phenotype was not informative (e.g. see F2 lineage X3_29 from cross 3, in which the two possible genotypes [*op2/CP2* or *CP1/CP2*] are expected to result in cyclical parthenogenesis) the two possible genotypes are mentioned (in that case the rectangle is white for the allele inherited from the female). Yet only the genotype corresponding to the left region (38.9 cM) is kept in Table 1 because both QTL and genome scan analyses suggest the causal locus is closer from marker 116879_10 (38.9 cM) than D_111865_3 (48.5 cM). Since the lineage (X3_4) used as mother for cross 9 recombined at position 49 cM, recombinant alleles are shown in red in this lineage and in its progeny.
(DOC)

Figure S2 Genome scans of wild OP and CP populations to identify outlier markers. Genetic differentiation (*F_CT_*) among six wild populations (∼18 ind/pop) experiencing selection for CP (those collected in North-east France and Switzerland) and selection for OP (those collected south-west France) as a function of heterozygozity for each of the 436 microsatellite loci estimated with ARLEQUIN 3.5. In this hierarchical analysis, populations were grouped according to reproductive strategy (three OP and three CP populations). The line represents the 99th quantile of the neutral envelope. Black dots: non outlier loci; yellow, orange and red dots represent outliers at α = 0.1, 0.05 and 0.01, respectively. Locus name is shown for the four 1% outliers.
(TIF)

Figure S3 Genetic diversity along chromosomes. Expected heterozygosity calculated over OP populations (pink line) and CP populations (black line) along chromosomes on a 15-cM sliding window is shown. The black bar shows the location of the 95% CI of the QTL for reproductive mode variation.
(TIF)

Table S1 Predicted functions of genes located on the three scaffolds that lay within the candidate region. Putative functions were inferred from comparisons to the NR peptide database (NCBI, July 2013 version) and based on the identification of protein domains using Interproscan 5 (Interpro database version of January 2014). Genes in bold are those referred to in the discussion. Scaffolds GL350218, GL350005 and GL350062, respectively, carry microsatellite markers D_116808_1 (located at 38.9 cM on the X chromosome), 116879_10 (39.1 cM) and D_111865_3 (48.5 cM), respectively.
(DOC)

Table S2 List of markers used in genetic mapping/QTL and genome scan approaches. Primer sequences are given in [42]–[44]. See [44] for PCR conditions.
(DOC)

Table S3 Geographical origin of populations used in the genome scan approach. The number of individuals used in the analyses is shown. All individuals were collected on *Medicago sativa* host plant. CP: cyclical parthenogenesis, OP: obligate parthenogenesis.
(DOC)

## References

[1] Neaves WB, Baumann P (2011) Unisexual reproduction among vertebrates. Trends Genet 27: 81–88. 21334090 10.1016/j.tig.2010.12.002

[2] Normark BB (2003) The evolution of alternative genetic systems in insects. Annu Rev Entomol 48: 397–423. 12221039 10.1146/annurev.ento.48.091801.112703

[3] Schön I, Martens K, van Dijk PJ (2009) Lost Sex – The Evolutionary Biology of Parthenogenesis; Schön I, Martens K, van Dijk PJ, editors. Berlin: Springer-Verlag.

[4] Bell G (1982) The Masterpiece of Nature: The Evolution and Genetics of Sexuality. Berkeley: University of California Press.

[5] Plantard O, Rasplus JY, Mondor G, Le Clainche I, Solignac M (1998) Wolbachia-induced thelytoky in the rose gallwasp *Diplolepis spinosissimae* (Giraud) (Hymenoptera : Cynipidae), and its consequences on the genetic structure of its host. Proc R Soc B-Biol Sci 265: 1075–1080.

[6] Delmotte F, Sabater-Munoz B, Prunier-Leterme N, Latorre A, Sunnucks P, et al (2003) Phylogenetic evidence for hybrid origins of asexual lineages in an aphid species. Evolution 57: 1291–1303. 12894937 10.1111/j.0014-3820.2003.tb00337.x

[7] Tucker AE, Ackerman MS, Eads BD, Xu S, Lynch M (2013) Population-genomic insights into the evolutionary origin and fate of obligately asexual *Daphnia pulex* . Proc Natl Acad Sci USA 110: 15740–15745. 23959868 10.1073/pnas.1313388110 PMC3785735

[8] Delmotte F, Leterme N, Bonhomme J, Rispe C, Simon JC (2001) Multiple routes to asexuality in an aphid species. Proc R Soc B-Biol Sci 268: 2291–2299. 10.1098/rspb.2001.1778 PMC1088879 11703868

[9] Simon JC, Delmotte F, Rispe C, Crease T (2003) Phylogenetic relationships between parthenogens and their sexual relatives: the possible routes to parthenogenesis in animals. Biol J Linnean Soc 79: 151–163.

[10] Dedryver CA, Le Gallic JF, Maheo F, Simon JC, Dedryver F (2013) The genetics of obligate parthenogenesis in an aphid species and its consequences for the maintenance of alternative reproductive modes. Heredity 110: 39–45. 22990313 10.1038/hdy.2012.57 PMC3522239

[11] Lynch M, Seyfert A, Eads B, Williams E (2008) Localization of the genetic determinants of meiosis suppression in *Daphnia pulex* . Genetics 180: 317–327. 18689898 10.1534/genetics.107.084657 PMC2535684

[12] Eads BD, Tsuchiya D, Andrews J, Lynch M, Zolan ME (2012) The spread of a transposon insertion in Rec8 is associated with obligate asexuality in *Daphnia* . Proc Natl Acad Sci USA 109: 858–863. 22215604 10.1073/pnas.1119667109 PMC3271927

[13] Lattorff HMG, Moritz RFA, Fuchs S (2005) A single locus determines thelytokous parthenogenesis of laying honeybee workers (*Apis mellifera capensis*). Heredity 94: 533–537. 15741997 10.1038/sj.hdy.6800654

[14] Sandrock C, Vorburger C (2011) Single-locus recessive inheritance of asexual reproduction in a parasitoid wasp. Curr Biol 21: 433–437. 21353557 10.1016/j.cub.2011.01.070

[15] Stelzer CP, Schmidt J, Wiedlroither A, Riss S (2010) Loss of sexual reproduction and dwarfing in a small metazoan. Plos One 5: e12854. 20862222 10.1371/journal.pone.0012854 PMC2942836

[16] Davis GK (2012) Cyclical parthenogenesis and viviparity in aphids as evolutionary novelties. J Exp Zool Part B 318B: 448–459. 10.1002/jez.b.22441 22644631

[17] Wilson ACC, Sunnucks P, Hales DF (1997) Random loss of X chromosome at male determination in an aphid, *Sitobion* near *fragariae*, detected using an X-linked polymorphic microsatellite marker. Genet Res 69: 233–236.

[18] Simon JC, Rispe C, Sunnucks P (2002) Ecology and evolution of sex in aphids. Trends Ecol Evol 17: 34–39.

[19] Simon JC, Stoeckel S, Tagu D (2010) Evolutionary and functional insights into reproductive strategies of aphids. C R Biol 333: 488–496. 20541160 10.1016/j.crvi.2010.03.003

[20] Dedryver CA, Hullé M, Le Gallic JF, Caillaud MC, Simon JC (2001) Coexistence in space and time of sexual and asexual populations of the cereal aphid *Sitobion avenae* . Oecologia 128: 379–388. 24549907 10.1007/s004420100674

[21] Rispe C, Pierre JS, Simon JC, Gouyon PH (1998) Models of sexual and asexual coexistence in aphids based on constraints. J Evol Biol 11: 685–701.

[22] Halkett F, Plantegenest M, Bonhomme J, Simon JC (2008) Gene flow between sexual and facultatively asexual lineages of an aphid species and the maintenance of reproductive mode variation. Mol Ecol 17: 2998–3007. 18466234 10.1111/j.1365-294X.2008.03798.x

[23] Halkett F, Kindlmann P, Plantegenest M, Sunnucks P, Simon JC (2006) Temporal differentiation and spatial coexistence of sexual and facultative asexual lineages of an aphid species at mating sites. J Evol Biol 19: 809–815. 16674577 10.1111/j.1420-9101.2005.01055.x

[24] Frantz A, Plantegenest M, Simon JC (2006) Temporal habitat variability and the maintenance of sex in host populations of the pea aphid. Proc R Soc B-Biol Sci 273: 2887–2891. 10.1098/rspb.2006.3665 PMC1664625 17015368

[25] Via S, Conte G, Mason-Foley C, Mills K (2012) Localizing F-ST outliers on a QTL map reveals evidence for large genomic regions of reduced gene exchange during speciation-with-gene-flow. Mol Ecol 21: 5546–5560. 23057835 10.1111/mec.12021

[26] Hawthorne DJ, Via S (2001) Genetic linkage of ecological specialization and reproductive isolation in pea aphids. Nature 412: 904–907. 11528477 10.1038/35091062

[27] Sloane MA, Sunnucks P, Wilson ACC, Hales DF (2001) Microsatellite isolation, linkage group identification and determination of recombination frequency in the peach-potato aphid, *Myzus persicae* (Sulzer) (Hemiptera : Aphididae). Genet Res 77: 251–260. 11486508 10.1017/s0016672301005018

[28] Wright S (1931) Evolution in mendelian populations. Genetics 16: 97–159. 17246615 10.1093/genetics/16.2.97 PMC1201091

[29] Mills LS, Allendorf FW (1996) The one-migrant-per-generation rule in conservation and management. Conserv Biol 10: 1509–1518.

[30] Rispe C, Pierre JS (1998) Coexistence between cyclical parthenogens, obligate parthenogens, and intermediates in a fluctuating environment. J Theor Biol 195: 97–110. 9802953 10.1006/jtbi.1998.0784

[31] Balloux F, Lehmann L, de Meeus T (2003) The population genetics of clonal and partially clonal diploids. Genetics 164: 1635–1644. 12930767 10.1093/genetics/164.4.1635 PMC1462666

[32] Paczesniak D, Adolfsson S, Liljeroos K, Klappert K, Lively CM, et al (2014) Faster clonal turnover in high-infection habitats provides evidence for parasite-mediated selection. J Evol Biol 27: 417–428. 24417476 10.1111/jeb.12310

[33] Muller HJ (1964) The relation of recombination to mutational advance. Mutat Res 106: 2–9. 14195748 10.1016/0027-5107(64)90047-8

[34] Maynard Smith J (1978) The evolution of sex: Cambridge University Press.

[35] Charlesworth B, Charlesworth D (1975) Experiment on recombination load in *Drosophila melanogaster* . Genet Res 25: 267–274. 810390 10.1017/s001667230001569x

[36] IAGC (2010) Genome sequence of the pea aphid *Acyrthosiphon pisum* . Plos Biol 8: e1000313. 20186266 10.1371/journal.pbio.1000313 PMC2826372

[37] Le Trionnaire G, Francis F, Jaubert-Possamai S, Bonhomme J, De Pauw E, et al (2009) Transcriptomic and proteomic analyses of seasonal photoperiodism in the pea aphid. Bmc Genomics 10: 456. 19788735 10.1186/1471-2164-10-456 PMC2763885

[38] Corbitt TS, Hardie J (1985) Juvenile hormone effects on polymorphism in the pea aphid, *Acyrthosiphon pisum* . Entomol Exp Appl 38: 131–135.

[39] Gallot A, Shigenobu S, Hashiyama T, Jaubert-Possamai S, Tagu D (2012) Sexual and asexual oogenesis require the expression of unique and shared sets of genes in the insect *Acyrthosiphon pisum* . Bmc Genomics 13: 76. 22336141 10.1186/1471-2164-13-76 PMC3313892

[40] Peccoud J, de la Huerta M, Bonhomme J, Laurence C, Outreman Y, et al (2014) Widespread host-dependent hybrid unfitness in the pea aphid species complex. Evolution 68: 2983–2995. 24957707 10.1111/evo.12478

[41] Ferrari J, Via S, Godfray HCJ (2008) Population differentiation and genetic variation in performance on eight hosts in the pea aphid complex. Evolution 62: 2508–2524. 18647340 10.1111/j.1558-5646.2008.00468.x

[42] Jaquiéry J, Rispe C, Roze D, Legeai F, Le Trionnaire G, et al (2013) Masculinization of the X chromosome in the pea aphid. Plos Genet 9: e1003690. 23950732 10.1371/journal.pgen.1003690 PMC3738461

[43] Jaquiéry J, Stoeckel S, Nouhaud P, Mieuzet L, Maheo F, et al (2012) Genome scans reveal candidate regions involved in the adaptation to host plant in the pea aphid complex. Mol Ecol 21: 5251–5264. 23017212 10.1111/mec.12048

[44] Jaquiéry J, Stoeckel S, Rispe C, Mieuzet L, Legeai F, et al (2012) Accelerated evolution of sex chromosomes in aphids, an X0 system. Mol Biol Evol 29: 837–847. 21998277 10.1093/molbev/msr252

[45] Hales DF, Wilson ACC, Sloane MA, Christophesimon J, Legallic JF, et al (2002) Lack of detectable genetic recombination on the X chromosome during the parthenogenetic production of female and male aphids. Genet Res 79: 203–209. 12220127 10.1017/s0016672302005657

[46] Green P, Falls K, Crooks S (1990) Documentation for CRIMAP, version 2.4.

[47] Voorrips RE (2002) MapChart: Software for the graphical presentation of linkage maps and QTLs. J Hered 93: 77–78. 12011185 10.1093/jhered/93.1.77

[48] Gilbert H, Le Roy P, Moreno C, Robelin D, Elsen JM (2008) QTLMAP, a software for QTL detection in outbred populations. Ann Hum Genet 72: 694–694.

[49] Simon J, Leterme N, Latorre A (1999) Molecular markers linked to breeding system differences in segregating and natural populations of the cereal aphid *Rhopalosiphum padi* L. Mol Ecol 8: 965–973. 10434417 10.1046/j.1365-294x.1999.00648.x

[50] Bacciu N, Bed'Hom B, Filangi O, Rome H, Gourichon D, et al (2014) QTL detection for coccidiosis (Eimeria tenella) resistance in a Fayoumi x Leghorn F-2 cross, using a medium-density SNP panel. Genet Sel Evol 46: 14. 24552175 10.1186/1297-9686-46-14 PMC3936936

[51] Kileh-Wais M, Elsen JM, Vignal A, Feves K, Vignoles F, et al (2013) Detection of QTL controlling metabolism, meat quality, and liver quality traits of the overfed interspecific hybrid mule duck. J Anim Sci 91: 588–604. 23148259 10.2527/jas.2012-5411

[52] Peccoud J, Ollivier A, Plantegenest M, Simon JC (2009) A continuum of genetic divergence from sympatric host races to species in the pea aphid complex. Proc Natl Acad Sci USA 106: 7495–7500. 19380742 10.1073/pnas.0811117106 PMC2678636

[53] Peel MC, Finlayson BL, McMahon TA (2007) Updated world map of the Koppen-Geiger climate classification. Hydrol Earth Syst Sci 11: 1633–1644.

[54] Excoffier L, Hofer T, Foll M (2009) Detecting loci under selection in a hierarchically structured population. Heredity 103: 285–298. 19623208 10.1038/hdy.2009.74

[55] Excoffier L, Lischer HEL (2010) Arlequin suite ver 3.5: a new series of programs to perform population genetics analyses under Linux and Windows. Mol Ecol Resour 10: 564–567. 21565059 10.1111/j.1755-0998.2010.02847.x

