# Supplementary figures and images for: Genetic Control of Contagious Asexuality in the Pea Aphid
Source: PLoS Genet. 2014 Dec 4;10(12):e1004838. doi: 10.1371/journal.pgen.1004838 (PMC4256089; doi:10.1371/journal.pgen.1004838)

**Supplementary Figure S1**


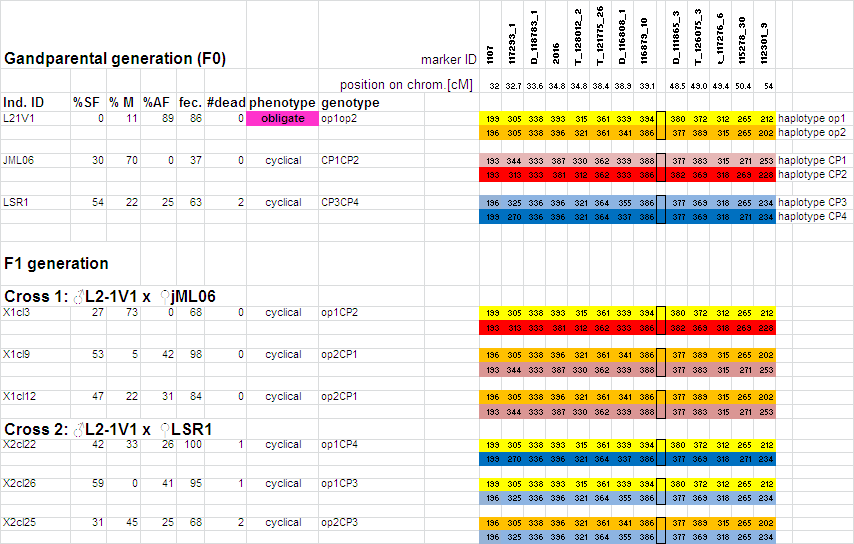


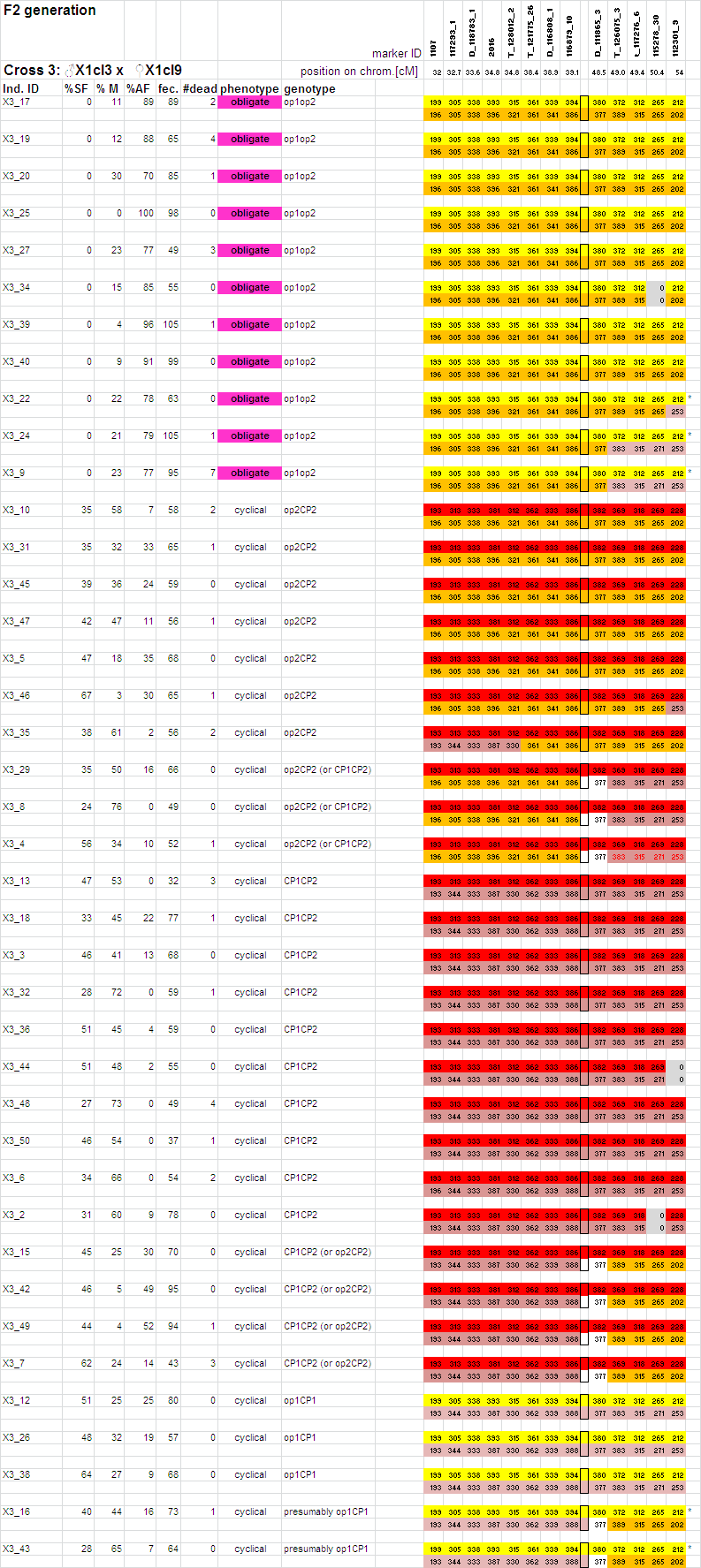


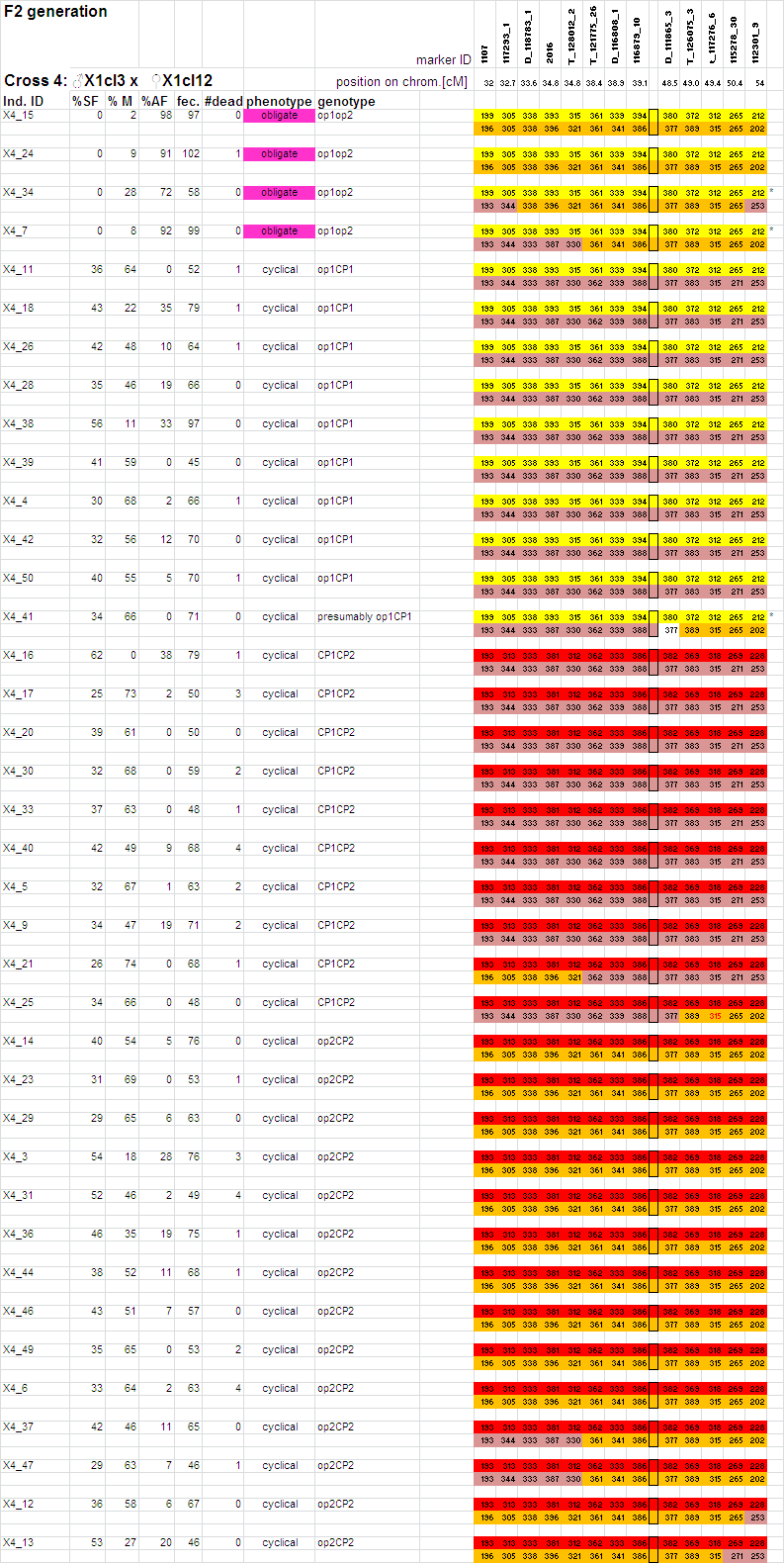


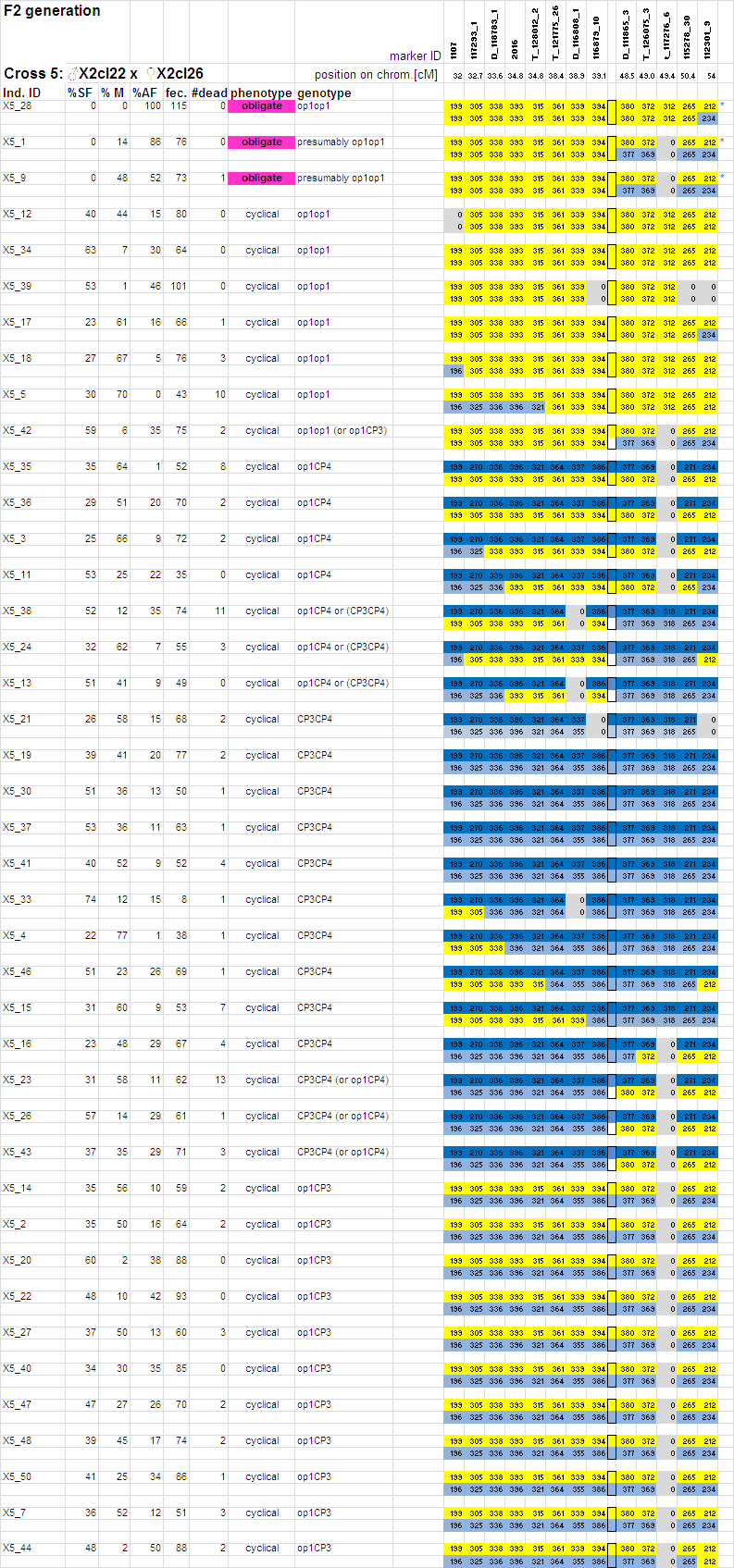


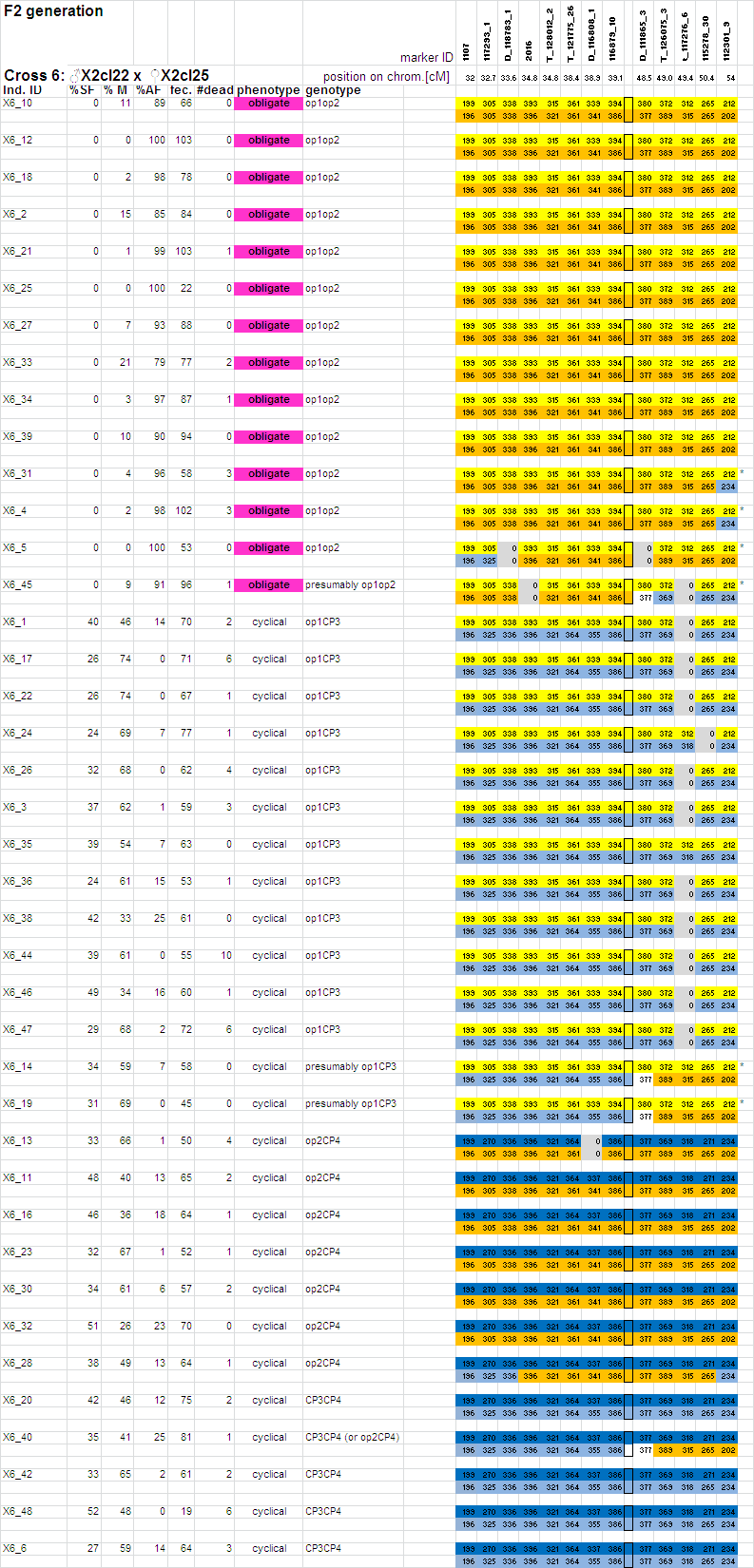


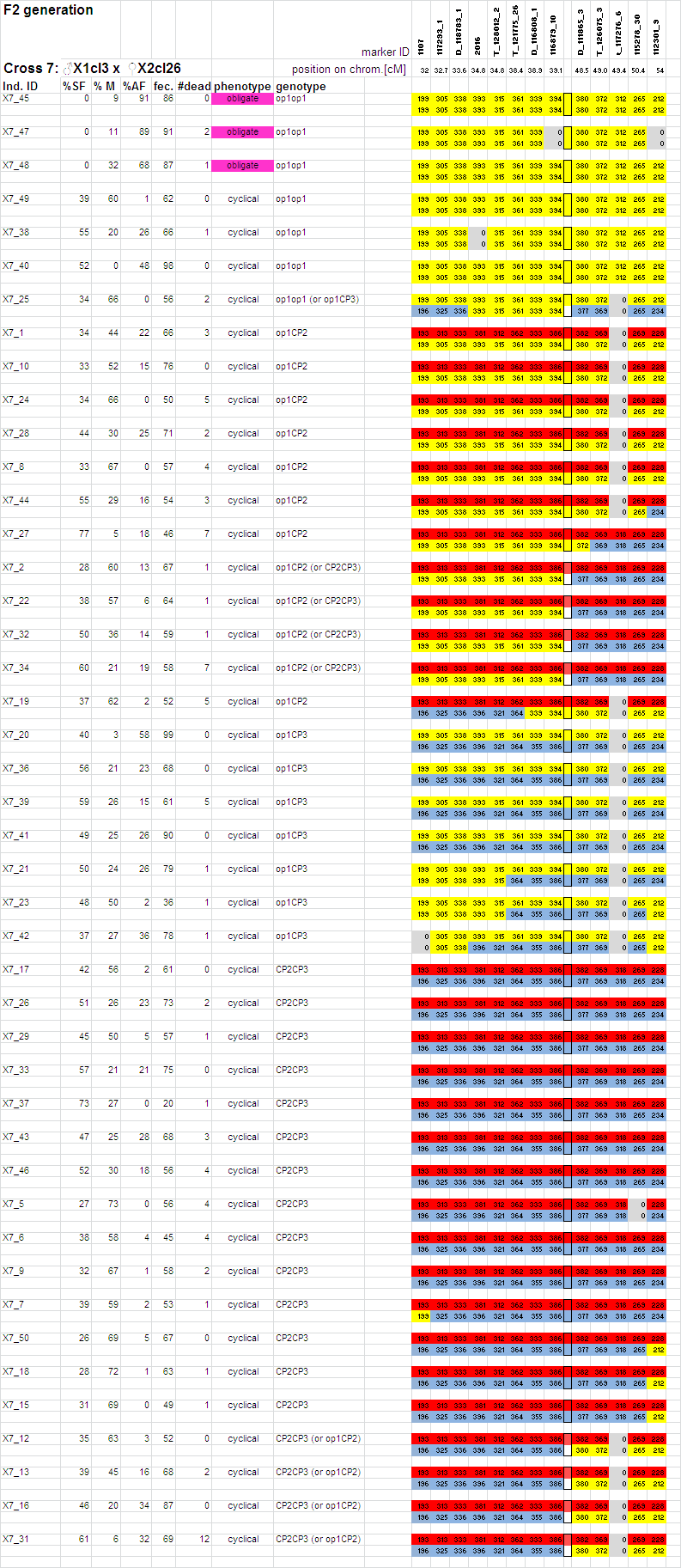


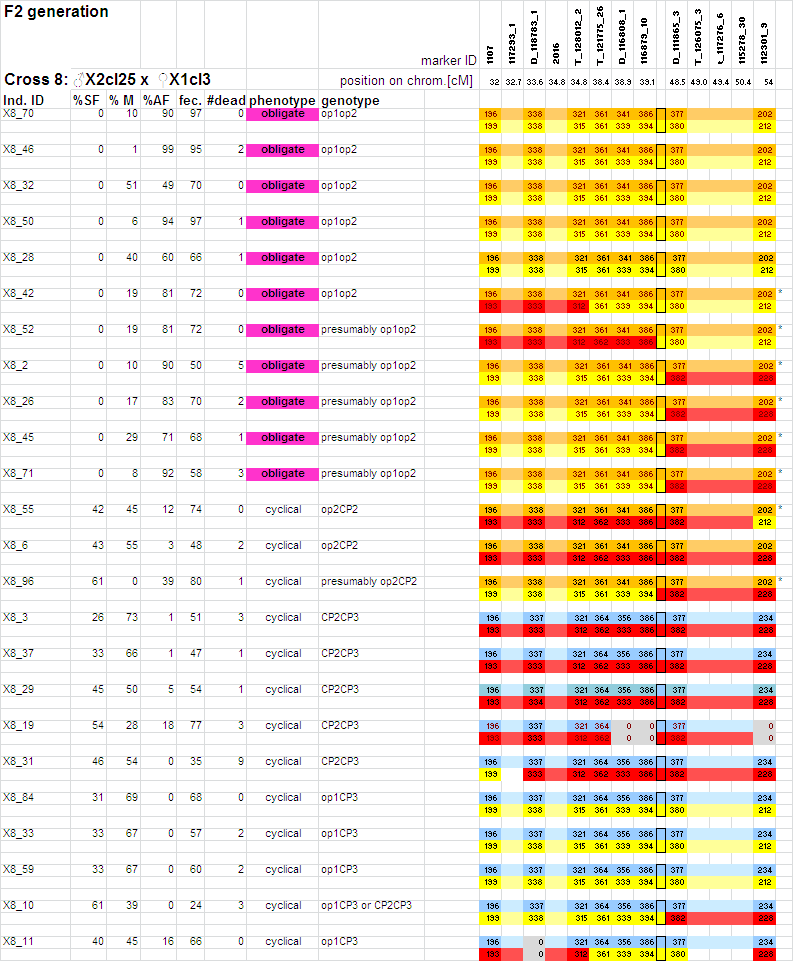


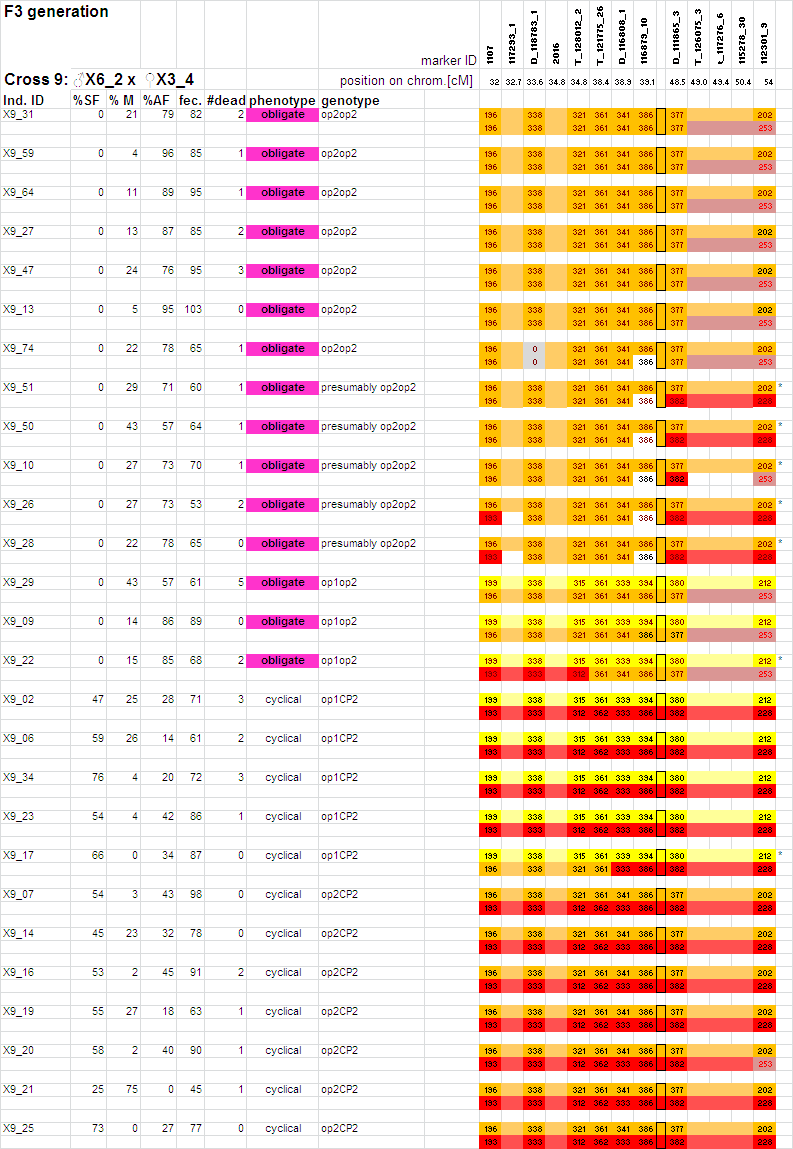

Supplement: Figure S1 — Phenotype and genotypic data from the 4-generation pedigree. For each of the 263 F0, F1, F2 or F3 lineage, we show the percentage of sexual females (SF), males (M) and asexual females (AF) produced when the lineage is placed in environmental conditions known to induce the production of sexual forms in cyclically parthenogenetic (CP) lineages. The fecundity (sum of the number of males, sexual females and asexual females produced) and the number of individuals that died before reaching the adult stage (i.e. that were not phenotyped) is also given. These values represent average over three replicates for each lineage (in all cases the three replicates gave the same response regarding the presence or absence of sexual females). Lineages were classified according to the production of sexual females (no sexual female produced: obligate parthenogenetic lineage [OP, showed in pink], sexual female produced: cyclical parthenogenetic lineage [CP]). We also show for each individual its genotype around the genomic region that contains the candidate locus for the production of sexual females (the 95% CI for the QTL ranges from ∼34 cM to ∼43 cM, and the portion of the X chromosome shown here span from 32 cM to 54 cM). Genotypes were phased and each of the different grandparental haplotype (op1, op2, CP1, CP2, CP3 and CP4) is shown with a different colour. The upper haplotype (in the F1 to F3 generations) corresponds to fragment of chromosome inherited from the father (i.e. without recombination since male aphids do not recombine). The lower haplotype was inherited from the mother (recombination might occur). Numbers correspond to the size of the allele at each microsatellite marker. Loci not successfully genotyped are indicated as 0 (in grey). Crosses 8 (F2) and 9 (F3) were performed after the identification of the candidate region, with the aims of further validation and to investigate the dominance of the two OP alleles (op1 and op2). Hence, only a subset of loci surrounding the [file pgen.1004838.s001.doc]

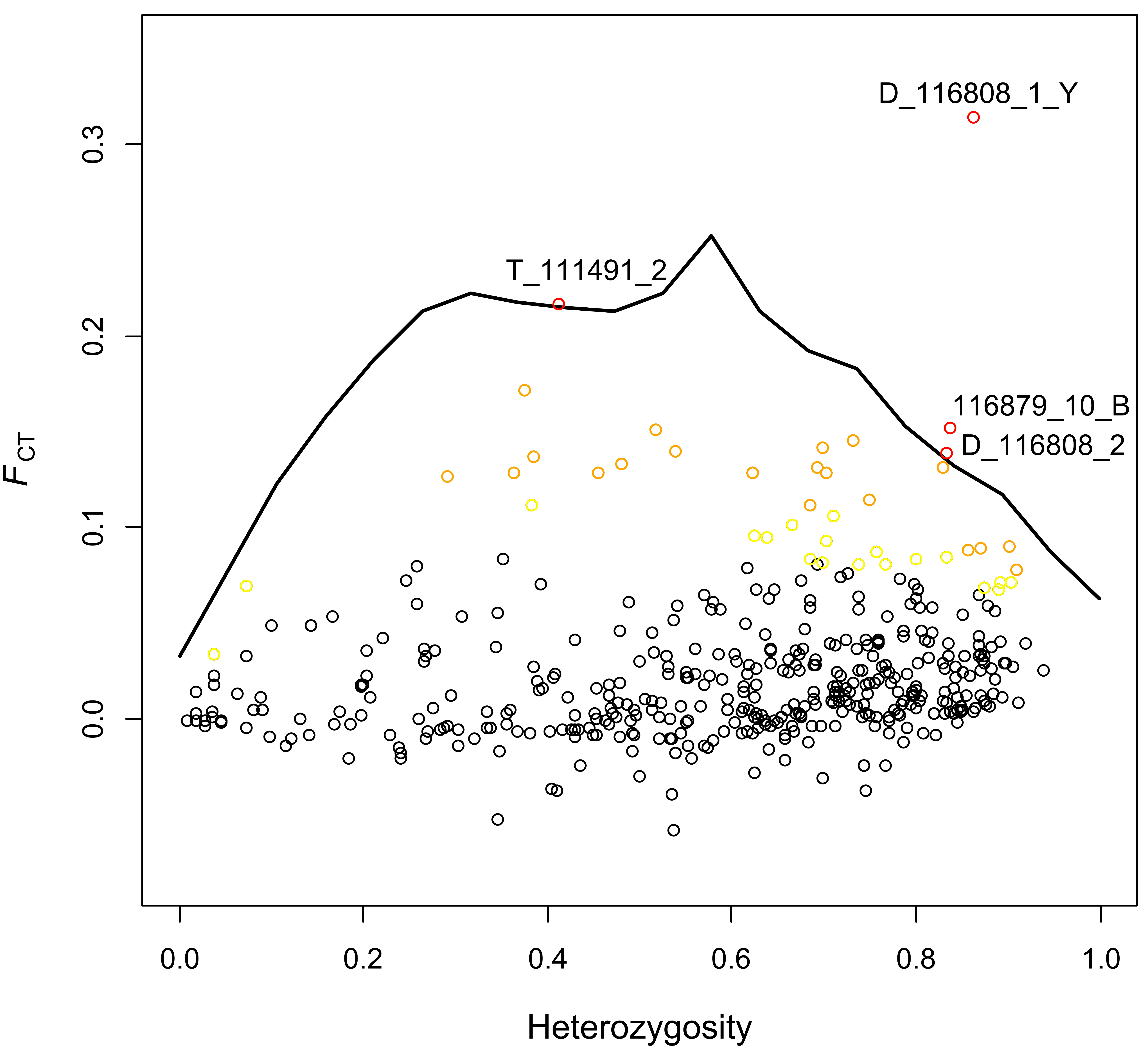

Supplement: Figure S2 — Genome scans of wild OP and CP populations to identify outlier markers. Genetic differentiation (FCT) among six wild populations (∼18 ind/pop) experiencing selection for CP (those collected in North-east France and Switzerland) and selection for OP (those collected south-west France) as a function of heterozygozity for each of the 436 microsatellite loci estimated with ARLEQUIN 3.5. In this hierarchical analysis, populations were grouped according to reproductive strategy (three OP and three CP populations). The line represents the 99th quantile of the neutral envelope. Black dots: non outlier loci; yellow, orange and red dots represent outliers at α = 0.1, 0.05 and 0.01, respectively. Locus name is shown for the four 1% outliers. (TIF) [file pgen.1004838.s002.tif]

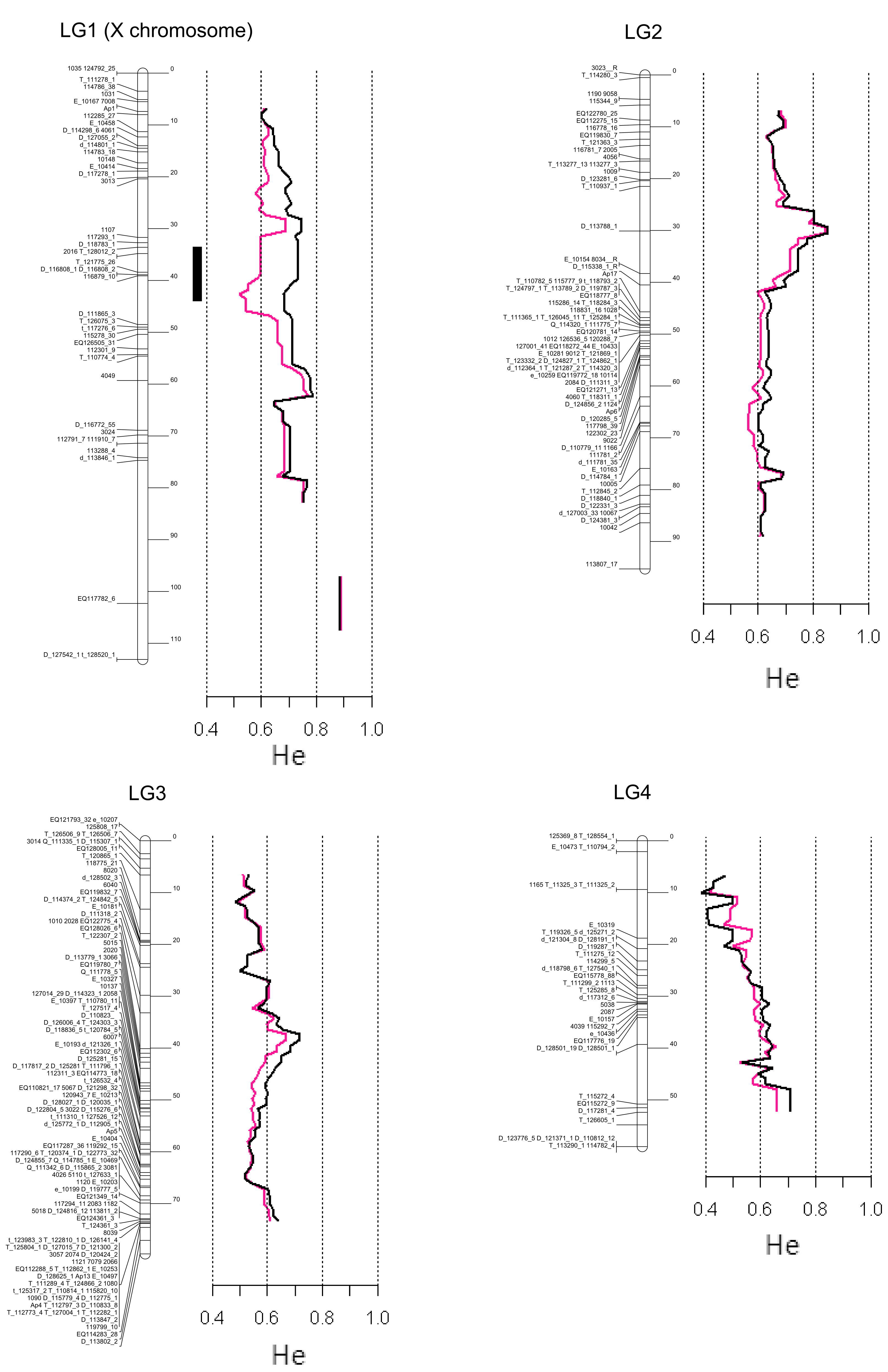

Supplement: Figure S3 — Genetic diversity along chromosomes. Expected heterozygosity calculated over OP populations (pink line) and CP populations (black line) along chromosomes on a 15-cM sliding window is shown. The black bar shows the location of the 95% CI of the QTL for reproductive mode variation. (TIF) [file pgen.1004838.s003.tif]
