# Supplementary material for: Genetic Control of Contagious Asexuality in the Pea Aphid
Source: PLoS Genet. 2014 Dec 4;10(12):e1004838. doi: 10.1371/journal.pgen.1004838 (PMC4256089; doi:10.1371/journal.pgen.1004838)
Supplement: Table S1 — Predicted functions of genes located on the three scaffolds that lay within the candidate region. Putative functions were inferred from comparisons to the NR peptide database (NCBI, July 2013 version) and based on the identification of protein domains using Interproscan 5 (Interpro database version of January 2014). Genes in bold are those referred to in the discussion. Scaffolds GL350218, GL350005 and GL350062, respectively, carry microsatellite markers D_116808_1 (located at 38.9 cM on the X chromosome), 116879_10 (39.1 cM) and D_111865_3 (48.5 cM), respectively. (DOC) [file pgen.1004838.s004.doc]

**Supplementary Table S1**

| **Scaffold ID** | **Gene ID** | **Beginning of gene** | **End of gene** | **Predicted function** |
| --- | --- | --- | --- | --- |
| GL350218 | ACYPI50894 | 1213 | 14286 | WD40 repeat-containing protein |
| **GL350218** | **ACYPI007673** | **17465** | **22079** | **Hydroxymethylglutaryl-CoA synthase** |
| **GL350218** | **ACYPI007080** | **26088** | **31180** | **Farnesyl-pyrophosphate synthase-like** |
| GL350218 | ACYPI003264 | 35292 | 42132 | Ubiquitin carboxyl-terminal hydrolase |
| GL350218 | ACYPI080530 | 43285 | 47615 | Uncharacterized protein |
| GL350218 | ACYPI005183 | 52634 | 62154 | Cyclin-dependent kinase 9-like |
| GL350218 | ACYPI43275 | 67744 | 81951 | Chromatin modification-related protein eaf3-like |
| GL350218 | ACYPI081672 | 85910 | 88015 | Uncharacterized protein |
| GL350218 | ACYPI062452 | 98930 | 114074 | Hypothetical protein |
| GL350218 | ACYPI081439 | 98930 | 114074 | Hypothetical protein |
| GL350218 | ACYPI42777 | 110357 | 113685 | Hypothetical protein |
| GL350218 | ACYPI069591 | 117586 | 131949 | WD repeat-containing protein |
| GL350218 | ACYPI089341 | 139834 | 140137 | Uncharacterized protein |
| GL350218 | ACYPI088869 | 145767 | 146436 | Uncharacterized protein |
| GL350218 | ACYPI085829 | 148965 | 150461 | Uncharacterized protein |
| GL350218 | ACYPI49308 | 155417 | 166390 | Hypothetical protein |
| GL350005 | ACYPI20100 | 1321 | 5480 | Uncharacterized protein |
| GL350005 | ACYPI071276 | 9604 | 17544 | Hypothetical protein |
| GL350005 | ACYPI20099 | 9916 | 10662 | Hypothetical protein |
| GL350005 | ACYPI087070 | 17706 | 18991 | Uncharacterized protein |
| GL350005 | ACYPI084577 | 114827 | 120138 | Uncharacterized protein |
| GL350005 | ACYPI087262 | 114827 | 120183 | Uncharacterized protein |
| GL350005 | ACYPI23795 | 123062 | 151345 | Hypothetical protein |
| GL350005 | ACYPI53193 | 157535 | 202750 | Uncharacterized protein |
| GL350005 | ACYPI006258 | 179585 | 223202 | Proton-coupled amino acid transporter like |
| GL350005 | ACYPI004320 | 242614 | 262825 | Amino acid transporter |
| GL350005 | ACYPI080526 | 261312 | 263265 | Amino acid transporter |
| GL350005 | ACYPI53198 | 282528 | 290381 | Amino acid transporter |
| GL350005 | ACYPI000536 | 297073 | 310216 | Amino acid transporter |
| GL350005 | ACYPI53200 | 316496 | 327068 | Sequence-specific DNA binding transcription factor |
| GL350005 | ACYPI082549 | 343157 | 349277 | Hypothetical protein |
| GL350005 | ACYPI006898 | 344855 | 347150 | Mitochondrial ornithine transporter |
| GL350005 | ACYPI005002 | 381578 | 392536 | Uncharacterized protein |
| GL350005 | ACYPI060729 | 388366 | 390442 | Hypothetical protein |
| **GL350005** | **ACYPI004011** | **412931** | **433424** | **Rhodopsin specific isomerase** |
| GL350005 | ACYPI005951 | 434678 | 449341 | Zinc finger |
| GL350005 | ACYPI081453 | 564865 | 570919 | Uncharacterized protein |
| GL350005 | ACYPI008382 | 581671 | 587243 | Similar to cytochrome c1 |
| GL350005 | ACYPI000104 | 590663 | 595760 | Similar to cytochrome c1 |
| GL350005 | ACYPI22867 | 602779 | 622468 | Dynein heavy chain |
| GL350005 | ACYPI23830 | 625726 | 627051 | Uncharacterized protein |
| GL350005 | ACYPI32274 | 637859 | 649743 | Zinc finger |
| GL350005 | ACYPI067233 | 650488 | 655994 | Uncharacterized protein |
| GL350005 | ACYPI29337 | 663385 | 664226 | Trypsin-like serine protease |
| GL350062 | ACYPI000975 | 22877 | 50063 | Calcium ion binding |
| GL350062 | ACYPI001611 | 76509 | 82954 | Fibroblast growth factor binding |
| GL350062 | ACYPI002668 | 68632 | 76515 | Leucine-rich repeat-containing protein |
| GL350062 | ACYPI002876 | 45134 | 53202 | Reeler domain |
| **GL350062** | **ACYPI003026** | **228935** | **232261** | **Insulin-like growth factor** |
| GL350062 | ACYPI003643 | 236260 | 247258 | Eukaryotic translation initiation factor kinase |
| GL350062 | ACYPI006233 | 306457 | 312133 | Tubulin beta-1 chain-like |
| GL350062 | ACYPI006778 | 106505 | 116904 | WD40 |
| GL350062 | ACYPI007099 | 154899 | 163038 | Helicase |
| GL350062 | ACYPI007463 | 212912 | 216763 | Uncharacterized protein |
| GL350062 | ACYPI009352 | 204126 | 206290 | Uncharacterized protein |
| GL350062 | ACYPI067639 | 101270 | 103293 | Hypothetical protein |
| GL350062 | ACYPI080731 | 291643 | 293886 | Uncharacterized protein |
| GL350062 | ACYPI080765 | 148216 | 149145 | Uncharacterized protein |
| GL350062 | ACYPI087453 | 117425 | 121720 | Uncharacterized protein |
| **GL350062** | **ACYPI41578** | **163032** | **200219** | **Cyclic nucleotide-gated cation channel beta (ROD photoreceptor)** |
| GL350062 | ACYPI44441 | 283329 | 291250 | Uncharacterized protein |
| GL350062 | ACYPI44442 | 267864 | 277093 | Protein binding |
| GL350062 | ACYPI44450 | 298466 | 304733 | Transmembrane GTPase |
| GL350062 | ACYPI44451 | 313516 | 315809 | Uncharacterized protein |
| GL350062 | ACYPI48731 | 741 | 2502 | Uncharacterized protein |
| GL350062 | ACYPI56665 | 118182 | 141688 | Histone lysine methylation |
