# Supplementary material for: Genetic Control of Contagious Asexuality in the Pea Aphid
Source: PLoS Genet. 2014 Dec 4;10(12):e1004838. doi: 10.1371/journal.pgen.1004838 (PMC4256089; doi:10.1371/journal.pgen.1004838)
Supplement: Table S2 — List of markers used in genetic mapping/QTL and genome scan approaches. Primer sequences are given in [42]–[44]. See [44] for PCR conditions. (DOC) [file pgen.1004838.s005.doc]

**Supplementary Table S2**

|  | Locus ID |
| --- | --- |
| Loci successfully used in both approaches (*n*=301) | 3023, T_114280_3, 1190, 9058, 115344_9, EQ122780_25, EQ112275_15, 116778_16, EQ119830_7, T_121363_3, 116781_7, 2005, 4056, T_113277_13, 113277_3, 1009, D_123281_6, T_110937_1, D_113788_1, E_10154, 8034, D_115338_1, T_110782_5, 115777_9, t_118793_2, T_124797_1, T_113789_2, D_119787_3, EQ118777_8, 115286_14, T_118284_3, 118831_16, 1028, T_111365_1, T_126045_11, T_125284_1, Q_114320_1, 111775_7, EQ120781_14, 1012, 126536_5, 120288_7, 127001_41, EQ118272_44, E_10433, E_10281, 9012__R, T_121869, T_123332_2, D_124827_1, T_124862_1, d_112364_1, T_121287_2, T_114320_3, e_10259, EQ119772_18, 10114, 2084, D_111311_3, EQ121271_13, 4060, T_118311_1, D_124856_2_R, 1124, Ap6, D_120285_5, 117798_39, 122302_23, 9022, D_110779_11, 1166, 111781_2, d_111781_35, E_10163, D_114784_1, 10005, T_112845_2, D_118840_1, D_122331_3, d_127003_33, 10067, D_124381_3, 10042, 113807_17, EQ121793_32, e_10207, 125808_17, T_126506_9, T_126506_7, 3014, Q_111335_1, D_115307_1, EQ128005_11, T_120865_1, 118775_21, 8020, d_128502_3, 6040, EQ119832_7, D_114374_2, T_124842_5, E_10181, D_111318_2, 1010, 2028, EQ122775_4, EQ128026_6, T_122307_2, 5015, 2020, D_113779_1, 3066, EQ119780_7, Q_111778_5, E_10327, 10137, 127014_29, D_114323_1, 2058, E_10397, T_110780_11, T_127517_4, D_110823_1, D_126006_4, T_124303_3, D_118836_5, t_120784_5, 6007, E_10193, d_121326_1, EQ112302_6, D_125281_15, D_117817_2, D_125281_17, T_111796_1, 112311_3, EQ114773_18, t_126532_4, EQ110821_17, 5067, D_121298_32, 120943_7, E_10213, D_128027_1, D_120035_1, D_122804_5, 3022, D_115276_6_G, t_111310_1, 127526_12, d_125772_1, D_112905_1, Ap5, E_10404, EQ117287_36, 119292_15, 117290_6, T_120374_1, D_122773_32, D_124855_7, Q_114785_1, E_10469, Q_111342_6, D_115865_2, 3081, 4026, 5110, t_127633_1, 1120, E_10203, e_10199, D_119777_5, EQ121349_14, 117294_11, 2083, 1182, 5018, D_124816_12, 113811_2, EQ128001_18, T_124361_3, 8039, t_123983_3, T_122810_1, D_126141_4, T_125804_1, D_127015_7, D_121300_2, 3057, 2074, D_120424_2, 1121, 7079, 2066, EQ112288_5, T_112862_1, E_10253, D_128625_1, Ap13, E_10497, T_111289_4, T_124866_2, 1080, t_125317_2, T_110814_1, 115820_10, 1090, D_115779_4, D_112775_1, Ap4, T_112797_3, D_110833_8_B, T_112773_4, T_127004_1, T_112282_1, D_113847_2, 119799_10, EQ114283_28, D_113802_2, 1035, 124792_25, T_111278_1, 114786_38, 1031, E_10167, 7008, Ap1, 112285_27, E_10458, D_114298_6, 4061, D_127055_2, d_114801_1, 114783_18, 10148, E_10414, D_117278_1, 3013, 1107, 117293_1, D_118783_1, 2016, T_128012_2_G, T_121775_26, D_116808_1, 116879_10, D_111865_3, T_126075_3, t_117276_6, 115278_30, EQ126505_31, 112301_9, T_110774_4, 4049, D_116772_55, 3024, 112791_7, 113288_4, d_113846_1, EQ117782_6, D_127542_1, t_128520_1, D_116808_2, 125369_8, T_128554_1, E_10473, T_110794_2, 1165, T_11325_3, T_111325_2, E_10319, T_119326_5, d_125271_2, d_121304_8, D_128191_1, D_119287_1, T_111275_12, 114299_5, d_118798_6, T_127540_1, EQ115778_88, T_111299_2, T_125285_8, d_117312_6, 5038, E_10157, 4039, 115292_7, e_10436, EQ117776_19, D_128501_19, D_128501_1, T_115272_4, EQ115272_9, D_117281_4, T_126605_1, D_123776_5, D_121371_1, D_110812_12, T_113290_1, 114782_4 |
| Loci successfully used only in the genome scan approach (*n*=136) | 1083, 1105, 112291_2, 1130, 117378_4, 117796_16, 119351_10, 120272_12, 121810_4, 2047, 3021, 3056, 4007, 4009, 4052, 5001, 6018, 6039, Ap18, d_110790_1, d_111280_1, D_112280_3, D_112776_11, D_114326_1, d_115785_2, D_115802_2, D_116897_1, d_116974_1, D_117328_2, D_117512_1, d_117781_10, D_119284_1, D_119783_3, D_119819_1, d_120801_1, D_121798_5, D_122777_2, D_122833_2, D_122967_2, D_123284_5, D_123314_1, D_123812_1, D_123838_1, D_124291_8, D_124353_2, D_126089_2, D_127007_10, d_127119_2, D_127502_15, D_127579_1, D_128013_10, D_128051_6, E_10156, E_10160, E_10339, E_10445, EQ111774_15, EQ114281_8, EQ115799_22, EQ118282_13, EQ119774_21, EQ121772_12, Q_116274_17, Q_121782_1, Q_123281_5, Q_124276_9, Q_124782_1, t_110797_1, T_111491_2, T_112277_6, T_112827_1, t_113792_9, T_115838_1, T_115843_27, T_117297_1, T_117781_9, T_117861_1, T_118790_29, t_119294_5, T_119302_1, T_119335_2, T_121390_2, T_121783_3, T_121814_1, T_123786_1, t_123796_4, T_125324_2, T_125832_2, T_127119_1, T_128006_1, 10017, 10024, 10084, 10139, 1060, 1141, 1168, 116830_45, 117272_16, 118300_1, 118780_11, 120277_6, 124284_11, 125279_9, 126044_9, 126104_4, 128093_26, 2033, 2056, 3004, 5086, 5093, 6019, 6058, 7006, 7023, 7047, 8002, 8008, 8035, 8041, 8045, 8046, 9010, D_117274_31, D_122315_1, D_122425_3, D_124278_8, D_124288_1, D_124381_9, D_128003_30, E_10435, Q_125794_1, T_111294_1, T_119335_1 |
| Loci successfully used only in the genetic mapping / QTL approach (*n*=4) | 1113, 2087, 111910_7, Ap17 |
