# Supplementary material for: Genetic Control of Contagious Asexuality in the Pea Aphid
Source: PLoS Genet. 2014 Dec 4;10(12):e1004838. doi: 10.1371/journal.pgen.1004838 (PMC4256089; doi:10.1371/journal.pgen.1004838)
Supplement: Table S3 — Geographical origin of populations used in the genome scan approach. The number of individuals used in the analyses is shown. All individuals were collected on Medicago sativa host plant. CP: cyclical parthenogenesis, OP: obligate parthenogenesis. (DOC) [file pgen.1004838.s006.doc]

**Supplementary Table S3**

| Population | Latitude | Longitude | # individuals |
| --- | --- | --- | --- |
| Switzerland (CP) | 46°28’ N | 6°26’ E | 17 |
| Ranspach (CP) | 48°01’ N | 7°33’ E | 18 |
| Mirecourt (CP) | 48°16’ N | 6°06’ E | 20 |
| Castelnaudary (OP) | 43°19’ N | 1°57’ E | 14 |
| Gers (OP) | 43°57’ N | 0°22’ E | 20 |
| Lusignan (OP) | 46°24’ N | 0°04’ E | 20 |
